# An Insight into the Metabolism of 2,5-Disubstituted Monotetrazole Bearing Bisphenol Structures: Emerging Bisphenol A Structural Congeners

**DOI:** 10.3390/molecules28031465

**Published:** 2023-02-02

**Authors:** Umesh B. Gadgoli, Yelekere C. Sunil Kumar, Deepak Kumar

**Affiliations:** 1 Department of Chemistry, M.S. Ramaiah University of Applied Sciences, Bengaluru 560054, Karnataka, India; 2 Dayanada Sagar Academy of Technology and Management, Kanakapura Rd, Opp. Art of Living International Centre, Udaypura, Bengaluru 560082, Karnataka, India

**Keywords:** bisphenol acetone, tetrazole bisphenol, in silico assessment, molecular dynamic simulations, CYP inhibition assay, substrate depletion assay, single-concentration Kobs assay, biotransformation pathway

## Abstract

The non-estrogenic 2,5-disubstituted tetrazole core-bearing bisphenol structures (TbB) are being researched as emerging structural congeners of Bisphenol A, an established industrial endocrine disruptor. However, there is no understanding of TbB’s adverse effects elicited via metabolic activation. Therefore, the current study aimed to investigate the metabolism of TbB ligands, with in silico results serving as a guide for in vitro studies. The Cytochrome P450 enzymes (CYP) inhibitory assay of TbB ligands on the seven human liver CYP isoforms (i.e., 1A2, 2A6, 2D6, 2C9, 2C8, 2C19, and 3A4) using human liver microsomes (HLM) revealed TbB ligand 223-3 to have a 50% inhibitory effect on all the CYP isoforms at a 10 μM concentration, except 1A2. The TbB ligand 223-10 inhibited 2B6 and 2C8, whereas the TbB ligand 223-2 inhibited only 2C9. The first-order inactivity rate constant (K${}_{\mathrm{obs}}$) studies indicated TbB ligands 223-3, 223-10 to be time-dependent (TD) inhibitors, whereas the TbB 223-2 ligand did not show such a significant effect. The 223-3 exhibited a TD inhibition for 2C9, 2C19, and 1A2 with K${}_{\mathrm{obs}}$ values of 0.0748, 0.0306, and 0.0333 min${}^{-1}$, respectively. On the other hand, the TbB ligand 223-10 inhibited 2C9 in a TD inhibition manner with K${}_{\mathrm{obs}}$ value 0.0748 min${}^{-1}$. However, the TbB ligand 223-2 showed no significant TD inhibition effect on the CYPs. The 223-2 ligand biotransformation pathway by in vitro studies in cryopreserved human hepatocytes suggested the clearance via glucuronidation with the predominant detection of only 223-2 derived mono glucuronide as a potential inactive metabolite. The present study demonstrated that the 223-2 ligand did not elicit any significant adverse effect via metabolic activation, thus paving the way for its in vivo drug–drug interactions (DDI) studies.

## 1. Introduction

Cytochrome P450 enzymes (CYP)-mediated metabolism is crucial in new chemical entity (NCE) research since their metabolic profile may influence many biological processes related to the liver that could potentially damage it. Such detrimental effects of an NCE, either in the clinical stage or after commercial use, could result in their withdrawal from the market due to poor metabolic profile. One such classic example of an industrial chemical banned from food contact applications because of its poor metabolic profile is Bisphenol A (BPA). BPA is an important industrial chemical belonging to the family of diphenyl alkanes and is a ubiquitous critical starting material for various polymers [1] such as polycarbonate, epoxy, and polysulfone, and as a chemical intermediate for producing various valuable materials.

**Figure 1:**
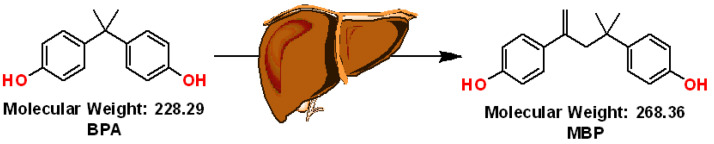
Image depicting metabolism of BPA to MBP in the liver based on the study of Yoshihara et al. [7]. A new unique potent BPA metabolite was observed having 40 mass units more than BPA and formed only in the presence of both cytosolic and microsomal fractions. The structure of this potent metabolite was established and named MBP, 4-methyl-2,4-bis (p hydroxyphenyl) pent-1-ene. Although BPA is a weak binder, its active MBP metabolite was several-thousand-fold more estrogenic than BPA.

BPA is known to leach into food products, and long-term exposure to it is known to be detrimental to health. It is an established endocrine disruptor and has the potential to induce hepatotoxicity via different mechanisms. Endocrine-mediated health effects such as reproductive toxicity, thyroid disruption, immunotoxicity, neurotoxicity, atopic diseases, and metabolic disorders have been reported as a result of BPA exposure [2]. Such toxic effects are ideally governed by the dose–response relationship between exposure to BPA and adverse health effects. The Consortium Linking Academic and Regulatory Insights on BPA Toxicity (CLARITY-BPA) program core study observed no negative impact on the health at the highest dose level of 25,000 μg kg bw${}^{-1}$ day${}^{-1}$, however, still at low-dose levels, some toxicological effects were detected on the reproductive system, brain, heart, and urinary tract. Hence, 2.5 μg (kg bw)${}^{-1}$ day${}^{-1}$ as the lowest-observed-adverse-effect level (LOAEL) was suggested [3]. The t-TDI was formerly set by EFSA in 2015 at 4 μg (kg bw)${}^{-1}$ day${}^{-1}$, however, it has recently submitted a draft opinion for a new TDI of 0.04 ng (kg bw)${}^{-1}$ day${}^{-1}$ [4]. Thus, there appears to be still some confusion, debate, and doubt regarding the low-dose effect coupled with the wide knowledge gap regarding BPA toxicity. Not just the BPA molecule itself, but also its bio-transformed compounds have raised health concerns. When orally administered to rats, BPA transformed into its glucuronide, ref. [5] which is non-estrogenic [6]. Yoshihara et al. [7] showed that the BPA’s estrogenicity multiplied after being incubated with the rat liver S9 fraction. They reported BPA-based catechol and o-quinone metabolites as CYP-mediated in rat liver S9 fraction. BPA-based o-quinone and catechol metabolites exhibited little activity, but a novel, potent BPA metabolite was discovered only when both cytosolic and microsomal fractions were present. This active metabolite’s structure was determined and named MBP, or 4-methyl-2,4-bis(p-hydroxyphenyl) pent-1-ene (Figure 1). Although BPA is a weak binder, its active MBP metabolite was several-thousand-fold more estrogenic than BPA. This potent metabolite is present in many animal species, including humans. Such an understanding has led to a cascade of research studies on the estrogenicity of BPA and its alternatives [8].

The common BPA replacements like bisphenol F (BPF), bisphenol AF (BPAF), bisphenol S (BPS), tetramethyl bisphenol F (TMBPF), Bisphenol S (BPS), Bisphenol Z (BPZ), Bisphenol M (BPM), etc., have been found to be exerting an endocrine-disrupting effect. Many of the metabolites formed by the biotransformation of BPA replacements were observed to be hydroxylated metabolites and electrophilic species during bioactivation in HLM and CYP isozymes, thus highlighting the need for their detailed toxicity and safety studies [9,10,11]. Therefore, if metabolism is the predominant route of elimination for a specific NCE, US Food and Drug Administration (US-FDA) [12,13] recommendations highlight the identification of metabolic pathways, principal metabolites, and potential drug–drug interactions. Hence, knowing the NCE’s metabolism is vital for their toxicological risk assessment.

**Figure 2:**
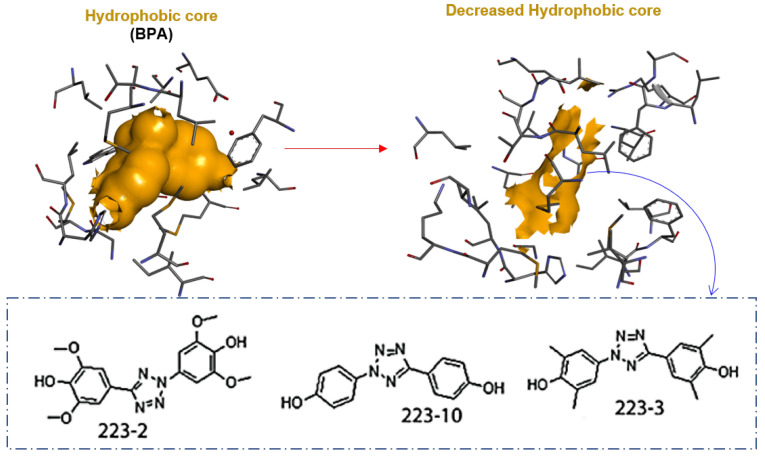
Recent research highlighted that substituting the BPA’s isopropylidene hydrophobic core with the tetrazole core (upper figure) decreased the estrogenicity of bisphenol. For example, two of the designed NCEs with bisphenol structures (223-2 and 223-3) bearing a tetrazole core (lower figure with chemical structures within the dotted rectangle) were potentially non-estrogenic by in vitro studies (Figure S1 of the Supporting Information) and, therefore, a privileged BPA replacement [14].

Our recent research on safer BPA replacements highlighted replacing the BPA’s isopropylidene core with a polar core. As a result, there was a decrease in bisphenol estrogenicity. For example, two of the designed NCEs with bisphenol structures (223-2 and 223-3) bearing tetrazole core (TbB) were potentially non-estrogenic by in vitro studies (Figure 2) [14]. It is hypothesized that the bisphenol with a polar core, due to its higher polarity, would be easily metabolized by the liver to a more polar compound, thus enabling safe excretion from the body. To our knowledge, there is no report on such a metabolism study on the TbB ligands, hence creating the need to understand their metabolism and adverse effect due to potential metabolic activation.

Therefore, the goal of the current work was to study the metabolism of three 2,5-disubstituted TbB ligands to get an insight into the potential adverse effects, if any, elicited via metabolic activation. Hence, two objectives were defined to accomplish this goal. The first was to understand the CYP inhibition potential of the 2,5-disubstituted TbB ligands by utilizing various in silico methodologies, such as structure- and ligand-based modeling, identifying the site of metabolism (SOM) and molecular dynamic simulations. The second objective was to perform the in vitro analysis in lieu of animal data to understand the inhibition potencies on various CYP isoforms, their time-dependent inhibition, and metabolic stability in human liver microsome (HLM), and cryopreserved human hepatocytes (CPHH) with an understanding for apparent intrinsic clearance, substrate depletion after incubation and T${}_{1/2}$. The completion of the objectives successfully enabled an understanding of the metabolic stability of the 2,5-disubstituted TbB ligands, their time-dependent (TD) potential, and the path of biotransformation. These studies highlighted one of the TbB ligands, 223-2, as a promising ligand with no potential adverse metabolic activation, thus paving the future for in vivo drug–drug interactions (DDI) studies.

## 2. Results and Discussion

Ideally, lipophilic compounds are converted into more hydrophilic metabolites in xenobiotic metabolism for enhanced excretion. Thus, an NCE entering humans via the oral route passes through the regions of first-pass metabolism, namely, the intestinal wall, followed by the liver. Since the liver is a detoxifying organ, it helps to eliminate these bio-transformed products through various enzymatic phase I and II pathways. On the contrary, xenobiotics or NCEs can also become adversely metabolized before reaching adequate plasma concentrations or inhibiting the enzymes. CYPs inhibitions lead to decreased elimination, thus causing xenobiotic/NCE-drug interactions and provoking adverse drug reactions. On the other hand, adverse metabolism can lead to the generation of reactive metabolites leading to hepatotoxicity. Therefore, it is imperative to gain insight into NCEs’ (such as TbB ligands of the current study) metabolic stability and CYP inhibition potential. Although there are many complexities in predicting metabolic stability and CYP inhibition, such an understanding can still be best gained computationally by parallel ligand and structure-based design approaches, as mentioned in the following sections.

### 2.1. Molecular Docking Study

The in silico insight into the molecular metabolism/inhibition of the TbB ligands was enabled by performing molecular docking studies. Ideally, in molecular docking, greater interest lies in optimizing the ligand and residue of the ligand binding pocket such that the ligand is bound tightly to the active site, leading to the desired pharmacological effect. However, in the case of docking to CYP, it is the opposite, wherein the interest is to avoid tight binding to decrease or minimize the binding affinity (BA) such that the enzyme does not become inhibited and undesired drug–drug interaction or toxicity is avoided. Therefore, to understand if the TbB ligands were potential inhibitors or non-inhibitors, they were docked into the active site of seven CYPs, namely, CYP2C9, CYP2B6, CYP2C8, CYP1A2, CYP2D6, CYP3A4, and CYP2C19, since the majority of these CYP represents ∼75% of drug metabolism [15]. The P450 CYP active site is relatively hydrophobic, and desolvation of the hydrophobic pocket of the heme environment is considered a significant contributor to binding affinity. As favourable and unfavourable contributions to the binding energy can be modeled using SeeSAR’s 12 (BioSolveIT Gmbh, Germany) hydrogen bond and dehydration (HYDE) energy scoring function, HYDE was used to perform predictive binding affinity calculations [16]. An insight into HYDE (Table S1 of the Supporting Information) and binding affinity is provided in the following ligand-CYP complexes.

#### 2.1.1. CYP2C9

**Figure 3:**
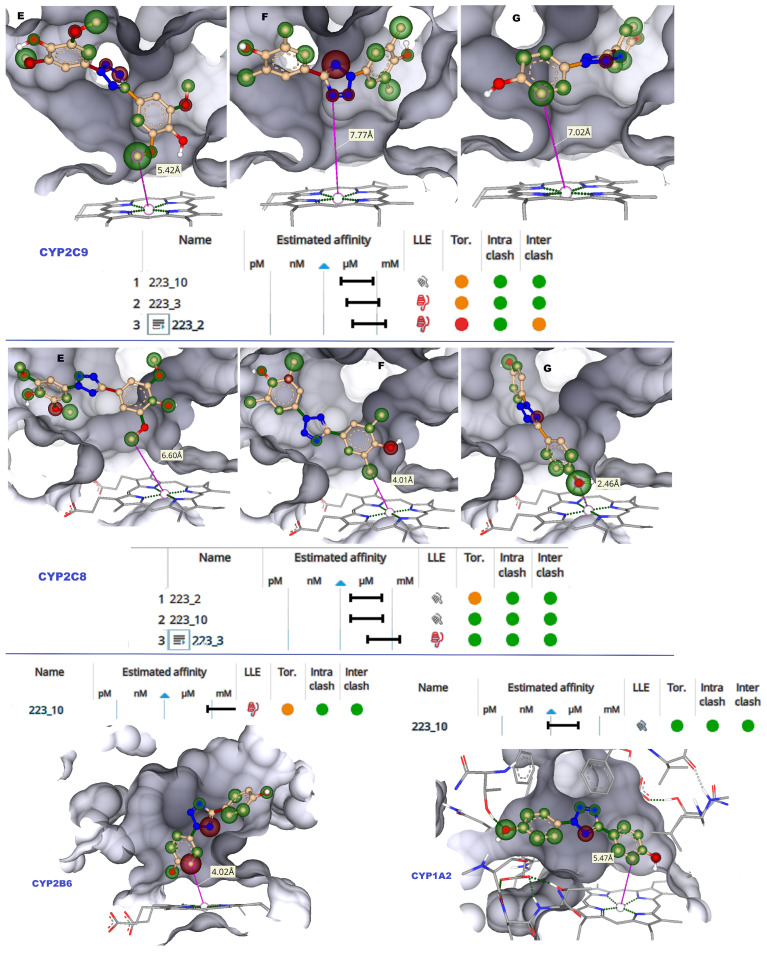
Images of CYP2C9, CYP2C8, CYP2B6, and CYP1A2 LBP docked with TbB ligands visualized with HYDE atomized coloring. White corona = neutrality, a green-colored corona = the ability of this atom to favorably contribute to BA, and a red-colored corona = the ability of this atom to non-favorably contribute to BA. (E) TbB ligand 223-2 atoms in LBP. (F) TbB ligand 223-3 atoms in LBP. (G) TbB ligand 223-10 atoms in LBP. The size of the corona sphere visually indicates the extent of favourable or non-favourable contribution to the BA. Larger spheres indicate more contribution and smaller spheres indicate less contribution to the BA. HYDE binding affinity (Ki) estimation of molecular docked poses of 223-2, 223-3, and 223-10 ligands in the active site of CYPs is represented as the range from mM < μM < nM < pM. Green circle = favourable to BA. Red circle = non-favourable to BA. BA = binding affinity.

CYP2C9 is the major enzyme driving phase I metabolism of xenobiotics, and undesirable drug–drug interaction or toxicity occur due to CYP2C9 inhibition [17]. The “6 Å rule” may be applied to study the effective binding to a CYP’s catalytic site. Such a rule means that the compounds present close to the heme iron, ideally within 6 Å, would oxidize the compound, acting as the site of metabolism [18]. The molecular docking results showed that 223-3 and 223-10 bind well to the active site of CYP2C9 (Figure S1 of the Supporting Information). In the case of 223-3 and 223-10, the distance of phenyl rings was beyond 6 Å from the heme iron, whereas in 223-2, the phenyl group was less than 6.0 Å, as depicted in Figure 3. The hydrogen bond contacts are vital for binding the ligands with CYPs. Hydrogen bond stabilized TbB 223-10 ligand with Thr 304 (1.8 Å). Further, the hydrophobic contacts with Ala 477, Thr 301, Ile 205, Glu 300, Ala 297, and Val 113 were observed with this ligand. The 223-3 ligand was stabilized with H-bond from Asn 107 (1.9 Å) and hydrophobically with Leu 208, Thr 301, Phe 114, Ile 205, and Ala 477. This ligand also had aromatic-aromatic interaction with Phe 114 (3.3 Å). The TbB ligand 223-2 formed H-bond with Asn 107 (2.1 Å). Additionally, it was stabilized hydrophobically with Leu 208, Ala 297, and Ile 205; however, it had a steric clash with Thr 301, Leu 362, and Val 237. It might be that such steric clash leading to non-bioactive conformation in the active site could be the potential reason for the least binding affinity of 223-2 when compared to others.

#### 2.1.2. CYP2C8

CYP2C8 has drawn much attention because of its crucial function in understanding clinically significant drug interactions. CYP2C8 has a relatively larger active site (1438 Å3), allowing it to accommodate larger xenobiotic substances [19]. It has carboxylic acid binding residues, Asn 99, Gly 98, and Ser 100 are located on the active site. Hence, the TbB ligand’s ability to inhibit CYP2C8 was focused on using molecular docking. The docked poses are presented in Figure S2 of the Supporting Information. The distance of phenyl rings in 223-3, and 223-10 docked poses were below 6 Å from the heme iron (Figure 3). In the case of 223-10, H-bonding is observed with Ala 297 (1.7 Å) and Ser 103 (2.1 Å). It had hydrophobic contacts with Thr 301, Hem 500, Ala 297, Ile 113, and Thr 107. The TbB ligand 223-3 formed H-bonds with Ser 100 (2.1 Å), Asn 217 (1.9 Å), and Val 296 (1.9 Å). It was found to be stabilized by hydrophobic interactions with Ile 102 and Ala 297. The TbB ligand 223-2 formed H-bond with Val 296 (1.9 Å), Ser 100 (2.0 Å), and Ser 103 (2.3 Å).

#### 2.1.3. CYP2B6 and 1A2

CYP2B6 is a highly polymorphic and inducible enzyme that plays a key role in human drug metabolism [20]. Variations in the function and expression of CYP2B6 significantly alter the pharmacokinetics and metabolism of many drugs. Therefore, potential clinical drug–drug interactions (DDI) arise from both genetic and pharmacological modulations of CYP2B6. Therefore, understanding the potential inhibition of CYP2B6 by TbB ligands is essential. In the case of TbB ligand 223-10, the distance of the phenyl ring was within 6.0 Å from the heme iron (Figure 3). The ligand 223-10 is stabilized by forming H-bond with Lys 479 (1.9 Å) and hydrophobic interactions with Thr 305, Glu 301, Phe 206, Phe 297, Leu 363, Ile 114, and Ala 298. The ligands 223-2 and 223-3 did not dock to the active site of CYP2B6.

To prevent pharmacokinetic interactions and to lessen or eliminate the harmful effects of toxicities mediated by xenobiotic metabolism, knowledge of the CYP1A2 inhibition by any xenobiotic is highly beneficial [21] Therefore, it is imperative to understand the inhibition of CYP1A2 by TbB ligands. In the active site of 1A2, the 223-10 phenyl ring was within 6.0 Å from the heme iron (Figure 3) and formed an H-bond with Thr 118 (2.2 Å). It formed hydrophobic contacts with Thr 321, Asp 320, Thr 118, Phe 260, Asn 312, Phe 125, Leu 497, and Ala 317. Additionally, it had aromatic–aromatic interactions with Phe 125 and 226. However, the ligands 223-2 and 223-3 did not dock to the CYP1A2 active site.

#### 2.1.4. CYP2D6

**Figure 4:**
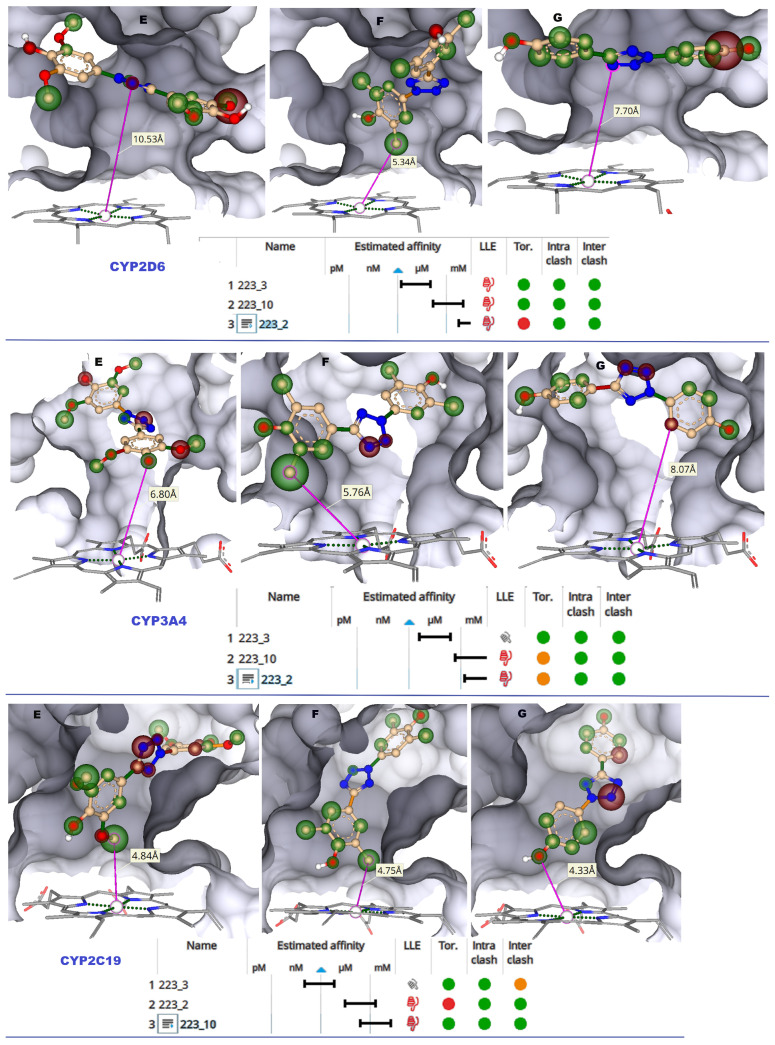
Images of CYP2D6, CYP3A4, and CYP2C19 LBP docked with TbB ligands visualized with HYDE atomized coloring. White corona = neutrality, a green-colored corona = the ability of this atom to favorably contribute to BA, and a red-colored corona = the ability of this atom to non-favorably contribute to BA. (E) TbB ligand 223-2 atoms in LBP. (F) TbB ligand 223-3 atoms in LBP. (G) TbB ligand 223-10 atoms in LBP. The size of the corona sphere visually indicates the extent of favourable or non-favourable contribution to the BA. Larger spheres indicate more contribution, and smaller spheres indicate less contribution to the BA. HYDE binding affinity (Ki) estimation of molecular docked poses of 223-2, 223-3, and 223-10 ligands in the active site of CYPs is represented as the range from mM < μM < nM < pM. Green circle = favourable to BA. Red circle = non-favourable to BA. BA = binding affinity.

CYP2D6 primarily metabolizes compounds containing basic amines such as aromatic amines and an aromatic planar ring due to acidic residues in the active site. The CYP2D6 is known to metabolize more than 20% of the drugs. Key residues such as Asp 301 and Glu 216 attract ligands to the active site [22]. Around 25% of all medications, including many antidepressants, rely on CYP2D6 activity for proper metabolism and effectiveness. Therefore, toxicity issues due to CYP2D6 inhibition by the TbB ligands are crucial to study. In the active site of 2D6 (Figure S3 of the Supporting Information), the TbB ligand 223-3 was observed to form H-bonding with Ser 304 (2.1 Å), and the phenyl ring was within 6.0 Å from the heme iron (Figure 4). It was additionally stabilized by the aromatic-aromatic interaction with Phe 483 and 120. It formed hydrophobic contacts with Phe 483, Glu 216, Thr 309, Val 308, Leu 213, Leu 484, Val 104, and Leu 121. The TbB ligand 223-10 formed H-bonding with Asp 301 (1.8 Å). Its atoms were beyond 6.0 Å from the heme iron. The TbB ligand 223-2 formed H-bonding with Gln 244 (1.9 Å), Ser 304 (1.8 Å), and Thr 375 (2.5 Å). Its atoms were beyond 6.0 Å from the heme iron.

#### 2.1.5. CYP3A4

CYP3A4 is a broad specificity oxygenase that metabolizes compounds of diverse structures. Its versatility in oxidizing bulky substrates is due to its considerably larger active site than any other CYP; hence, it is responsible for metabolizing more than 50% of the drugs in the market [23]. Therefore, it is considered a significant locus for problems with drug–drug interactions leading to many hospitalizations and deaths [24]. A better understanding of CYP3A4 inhibition by TbB ligands is, therefore, critical. The TbB ligand 223-10 is stabilized by Leu 483 (2.1 Å), Phe 13 (2.0 Å), and Ser 119 (2.1 Å) as shown in Figure S4 of the Supporting Information. It is also stabilized by aromatic–aromatic interaction with Phe 215 and Phe 204. The 223-3 ligand forms an H-bond with Arg 212 (2.0 Å), Ile 369 (2.3 Å), and Arg 106 (1.8 Å). Its phenyl group is within 6.0 Å from the heme iron (Figure 4). The TbB ligand 223-2 does not form an H-bond with active site residues, however, it has an aromatic–aromatic interaction with Phe 215 and steric clash with Phe 57 and Arg 372.

#### 2.1.6. CYP2C19

In the case of CYP2C19 active site (Figure S5 of the Supporting Information), H-bond formation is observed between TbB ligand 223-10 and Leu 102 (2.1 Å). The ligand is stabilized by aromatic–aromatic interactions with Phe 114. The phenyl group of all the three TbB ligands were within 6.0 Å from heme iron (Figure 4). The 223-3 ligand forms H-bond with Asn 204 (1.9 Å). It is stabilized by the aromatic–aromatic contact with Phe 114. In the case of 223-2, the H-bond is formed with Asn 107 (2.1 Å) and is additionally stabilized by aromatic–aromatic interaction Phe 476.

The molecular docking and HYDE prediction provided insightful information on how avidly the ligands bind to a given P450 enzyme. Molecular docking suggested that in some of the CYP, the atoms of some poses are within the 6 Å of the heme iron; however, to corroborate prediction of the metabolism site (SOM), which is key to understanding the ligand’s metabolic stability or instability, the additional computational tools were utilized such as DFT (Table S2 and Figure S6 of the Supporting Information), the ADMET Predictor Metabolism module [25] and XenoSite Metabolism and Reactivity Prediction Web Server [26].

### 2.2. Sites of Metabolism

XenoSite Cytochrome P450 Prediction Models predict atoms on a molecule that a specific CYP enzyme could likely oxidize. XenoSite provides visual output wherein a color gradient labels the potential SOMs, referring to the atom in a molecule that is metabolically labile and prone to metabolic reactions. For efficient visualizations of metabolism in a molecule, the major SOM is identified based on the color coding of the probability scale. In the default mode “background,” blue, white, and red colors are used. A zero probability of metabolism is represented by blue, a probability equal to the background probability of observing a SOM at random is represented by white, and a probability of 1.0 is indicated by red. Computational modeling graphical outputs highlighting metabolic reactivity and SOMs for the TbB ligands are displayed as a metabolic landscape in Figures S7–S9 of the Supporting Information. This output indicates the metabolic liability of each site. From the metabolic landscape shown in Figure S7 of the Supporting Information, the ligand 223-2 is predicted to be metabolized by 1A2, 2C19, 2C9, and 2C8 CYP450 isoenzymes, primarily in the methoxy groups shown with higher color scale. Similarly, in ligand 223-3, the substituted methyl groups are observed to be the potential SOMs (Figure S8 of the Supporting Information) but in ligand 223-10, though the carbon atoms of the phenyl rings are indicated to be labile, the likelihood is less due to the lower color scale (Figure S9 of the Supporting Information).

CYP’s are known to catalyze the oxidation of bisphenols, for example, BPA and, in one instance, as reported by Atkinson A. et al. [27,28]. BPA is metabolized to a DNA-reactive bisphenol-o-quinone adduct. Such reactive adducts might cause modifications of the code and gene dysregulation for DNA, while protein adducts can induce harmful immune responses and interfere with normal biological functions. Such probable metabolites as a result of metabolic reactions are directly related to SOM. An insight into the identification of the SOM and DNA adducts is, therefore, important for understanding the role of TbB ligands in adductive reactions and, consequently, their potential role in biological dysfunction, toxicity, and cancer risk. Hence, an online XenoSite reactivity model was employed to determine the labile electrophilic sites on atoms of TbB ligands susceptible to DNA, cyanide, GSH, and protein. The DNA model predicted a low probability of reactive intermediate formation as indicated by a lower scale presented in Figure S10 of the Supporting Information. The metabolic landscape in Figures S7–S9 of the Supporting Information for TbB ligands indicated the metabolic lability of each site by CYP enzymes, thus guiding the prediction of TbB ligands metabolites. Understanding their biotransformation pathway in humans enables predicting the potential metabolites formed and determining their potential toxicological hazard. Subsequently, ADMET Predictor Metabolism Module was used to predict the potential metabolites formed and their in silico estrogenicity using in-built AR and ER models (Figures S11–S13 and Table S3 of the Supporting Information).

### 2.3. Molecular Dynamic Simulations

From the molecular docking studies discussed in the previous section, it is understood that the TbB ligands show two binding motifs wherein, in a certain CYP binding pocket, there is direct interaction with heme iron, and in others, there is interaction with amino acids in the hydrophobic distal pocket without coordinating the heme iron. Since the molecular docking is considered static and to better understand the steadiness and conformation stability of the three TbB ligand-CYP complexes, 50 ns MD simulations were performed. The trajectories of MD simulations were studied to determine the accessibility and binding of the TbB ligand in each of the CYP isoforms.

#### 2.3.1. 223-2

**Figure 5:**
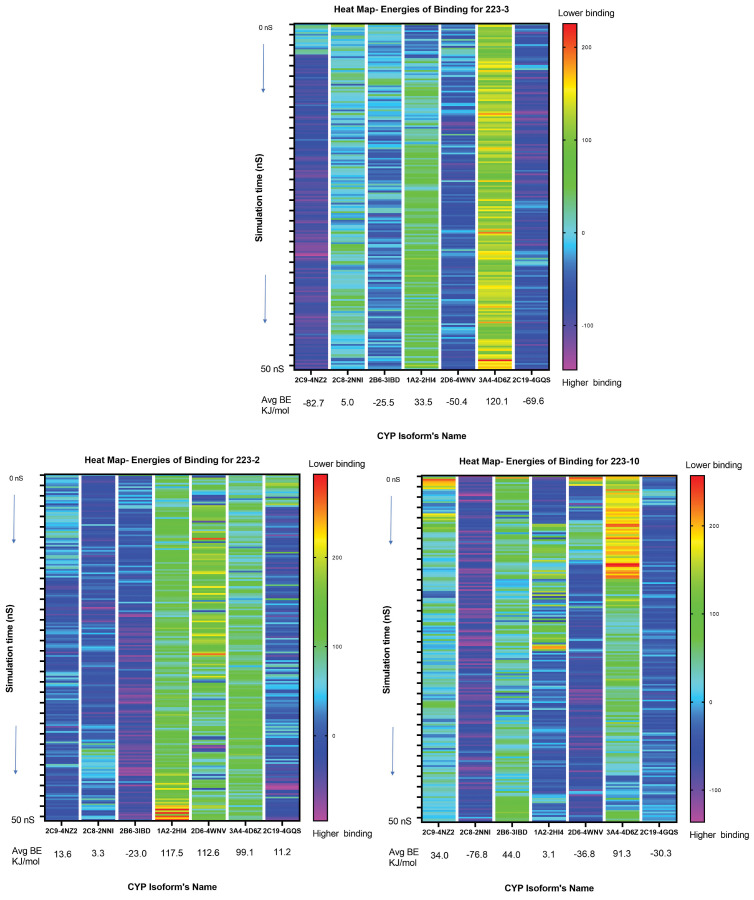
Binding energy of ligands is shown as a heat map calculated during the molecular dynamic (MD) simulation of 50 ns. The gradient of colored bands represents BE. The least binding is indicated by red bands, and the strongest binding by purple-colored bands. The greater the negative value of −ΔG, the better the stability of the ligand-CYP complex, and conversely, +ΔG means an unstable ligand-CYP complex. Based on the MD simulation, the binding of the ligands can be hypothesized as 223-3 > 223-10 > 223-2. Therefore, 223-2 is the least prominent binder.

After the completion of MD simulations, the relative binding free energy (BE) of the three TbB ligands was analyzed, as the heatmap shown in Figure 5. The data predicted 223-2 to have a weak binding to 1A2, 2D6, 3A4, 2C19, 2C9, and 2C8 CYP isoforms whereas strong binding was observed for 2B6. To gain more understanding on the reasons for weak and strong binding, interactions due to per residue contact were analyzed. The 223-2-1A2 and 223-2-2B6 CYP complexes data are provided in the Table 1 as representative example. Further, analysis of ligand movement RMSD (Figures S14–S17 of the Supporting Information) after superposing on the receptor revealed that the 223-2 ligand movement is dynamic and not stable in the 2B6 CYP binding pocket. There is a significant change in the RMSD, initially from 1.25 Å to 3.75 Å. Likewise, there is a change in RMSD of the ligand atoms over time, measured after superposing on the reference structure of the ligand. Thus, such ligand movement is assumed to cause stronger binding of the 223-2 ligand with the binding site residues, as witnessed in the free energy of the binding heat map (Figure 5) and could be the reason for strong binding. However, such observation is not witnessed in the other 223-2 CYP complexes and is found to be stable.

#### 2.3.2. 223-3

The free energy of the binding heat map presented in Figure 5 predicted 223-3 to have a weak binding to 3A4, 1A2, and 2C8 CYP isoforms, whereas strong binding was observed for 2C9, 2C19, 2D6, and 2B6. To understand better the possible reasons for weak and strong binding, interactions due to per residue contact were analyzed. The 223-3-2D6 and 223-3-3A4 CYP complexes data is provided in the Table 2 as representative example. The analysis of ligand movement RMSD (Figures S14–S17 of the Supporting Information) after superposing on the receptor revealed that the 223-3 ligand movement is unstable until 6 ns in the 3A4 CYP binding pocket, and there is a significant change in the RMSD only after 46 ns from 1.25 Å to 3.85 Å. Likewise, 223-3 ligand movement in 2D6 CYP binding pocket is unstable until 6 ns. It stabilizes after 6 ns at 6.0 Å until the end and there is no change in RMSD of the ligand atoms over time until 46 ns, measured after superposing on the reference structure of the ligand. Hydrogen bonding interactions from Ser 304 is hypothesized to be a contributor to stronger binding.

#### 2.3.3. 223-10

The free energy of the binding heat map presented in Figure 5 predicted 223-10 to have a weak binding to 1A2 and 3A4 CYP isoforms, whereas strong binding was observed for 2C8, 2D6, and 2C19. Such different interactions were understood by analyzing the per-residue contact. Hydrophobic interactions from sixteen residues stabilized the 223-10 2C8 CYP complex having an average contact of 57% per residue. In the case of CYP2D6, hydrophobic interactions from twenty-three residues stabilized this ligand binding complex with an average contact of 32% per residue apart from the hydrogen bonding interactions resulting from five residues with an average contact of 5% per residue. In the case of the 223-10 2C19CYP complex, twenty-three residues are participating with 31% hydrophobic interactions, 15% hydrophobic+hydrogen bond, and 5% hydrogen bonding interactions from the three residues. The analysis of ligand movement RMSD (Figures S14–S17 of the Supporting Information) after superposing on the receptor revealed that the 223-10 ligand movement is not stable in the 2C19CYP binding pocket. In the 2D6CYP binding pocket, 223-10 ligand movement was found to be stable, and in the 2C8CYP binding pocket, 223-10 ligand movement varied significantly. Maybe such ligand movement could be the reason for the stronger binding of the 223-10 in the CYP ligand binding pockets.

### 2.4. CYP Inhibitory Assay

**Figure 6:**
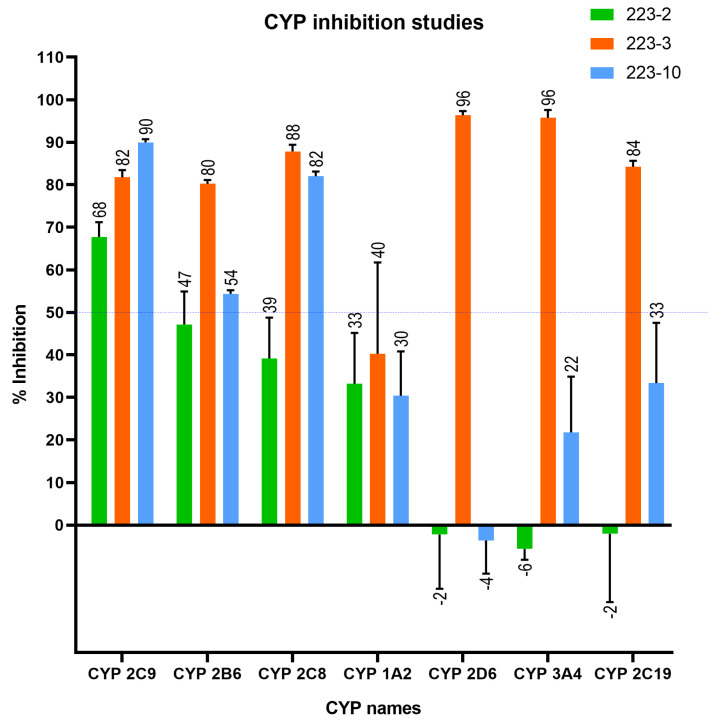
Inhibition assay results of TbB ligands by human CYP involving most common isoforms and their respective probe substrates at a single concentration of 10 μM (n = 3). The TbB ligand 223-3 was observed to have an above 50% inhibitory effect on all the CYP isoforms except CYP1A2. The TbB ligands 223-2, 223-3, and 223-10 exhibited an inhibitor effect of above 50% on CYP2C9. Based on the CYP inhibition assay, inhibiting ligands can potentially be ranked as 223-3 > 223-10 > 223-2. Therefore, 223-2 is the less prominent inhibitor.

The regulatory bodies and pharmaceutical firms are quite concerned about CYP inhibition. Therefore, medicinal chemists often change the lead structure to decrease CYP inhibition. If left uncorrected, it can cause DDIs, enhanced drug concentration, and, thus, toxicity. Hence, to understand the CYP inhibition, the in vitro metabolism of TbB ligands by human CYP involving most common isoforms such as CYP2C8 (Amodiaquine), CYP2D6 (Dextromethorphan), CYP2C9 (Tolbutamide), CYP2B6 (Bupropion), CYP3A4 (Midazolam), CYP1A2 (Phenacetin) and CYP2C19 (S-Mephenytoin) using their respective probe substrates at single concentration of 10 μM was investigated as shown in Figure 6.

The TbB ligands 223-2, 223-3, and 223-10 exhibited an inhibition effect of above 50% on CYP2C9, whereas only 223-3, 223-10 exhibited inhibition on CYP2B6 and CYP2C8. The TbB ligand 223-3 was observed to have an above 50% inhibitory effect on all the CYP isoforms except CYP1A2. Thus, the potential clinical interaction between 223-3 and these CYPs would be significant. The possible reason for such observation could be that the binding site of metabolizing enzymes is generally lipophilic; hence, these enzymes more readily accept lipophilic molecules. Since 223-3 is comparatively more lipophilic than 223-2 and 223-10 (Table S1 of the Supporting Information), hence might have a better binding affinity. Currently, there is no understanding of the TbB ligands stability in HLM and hepatocytes, hence, a substrate depletion assay was performed to have a better insight on the same.

### 2.5. Substrate Depletion Assay of TbB Ligands with HLM and Cryopreserved Hepatocytes

Currently, an understanding of metabolic stability of the three 2,5-disubstituted TbB ligands is unavailable. Such an understanding using a substrate depletion assay is required to comprehend their toxicological profile. Consequently, investigations using microsomes together with hepatocytes were carried out to obtain more thorough information to understand the different clearance pathways by CYP or non-CYP enzymes. This approach is also very valuable as it allows the investigation of the total cytochrome P450-mediated metabolism of TbB ligands without the need for complete knowledge of the metabolites. Further, it enables understanding the enzyme kinetics since this approach encompasses all the activity of the CYPs and all the biotransformation pathways involved in the metabolism.

**Figure 7:**
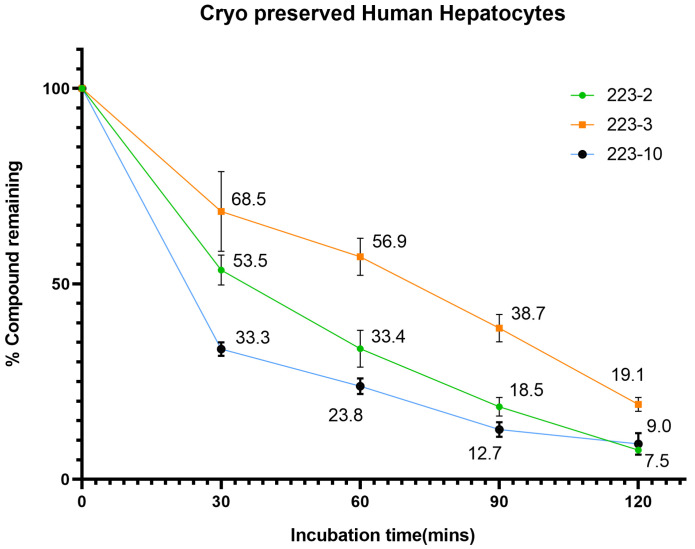
Metabolic stability studies of TbB ligands in hepatocytes—all three TbB ligands metabolized in cryopreserved hepatocytes (n = 3). Only <20% of the parent compound remained, suggesting that these ligands predominantly undergo Phase II metabolism or non-CYP pathways.

Microsomal stability was determined for the TbB ligands by monitoring the presence of compound after incubation in CPHH and HLM, as shown in Figure 7 and Figure S18 of the Supporting Information. Figure S18 of the Supporting Information shows that the phase 1 enzymes of HLM metabolizes the TbB ligand 223-3, whereas the other TbB ligands demonstrated stability in the HLM. Such an observation with NCEs is common because, with HLM, NADPH is used as a cofactor, thereby focusing mainly on phase 1 oxidation pathways such as P450 metabolism. Although additional enzymes, such as UDP-glucuronosyltransferase (UGTs), are present in HLM, the lack of the associated necessary cofactors prevents them from being functionally active (e.g., uridine diphosphate glucuronic acid (UDPGA)). In contrast, all the three TbB ligands metabolized in cryopreserved hepatocytes (Figure 7). Only <20% of the parent compound remained, suggesting that these ligands predominantly undergo phase II metabolism or non-CYP pathways. The reason could be that hepatocytes have all the necessary enzymes and cofactors for the metabolic processes. Since they contain the complete set of oxidative/reductive, hydrolytic, and conjugative drug metabolizing enzymes found in the liver, they are regarded as the gold standard. Therefore, they are preferred in vitro options for expressing metabolic stability as substrate depletion, total metabolic clearance, and T${}_{1/2}$ [29].

From the plot shown in Figure S18 of the Supporting Information, it can be inferred that the TbB ligands undergo slow biotransformation in HLM. Such high metabolic stability may result in drug interactions leading to toxicity of the parent compound. On the contrary, TbB ligands are undergoing rapid biotransformation in CPHH (Figure 7), and this may lead to generation of metabolites that are either toxic, active, or nonactive. Metabolic stability is typically expressed by in vitro half-life T${}_{1/2}$ and intrinsic clearance (CLint) terms. Intrinsic clearance (CLint) is measured using the substrate depletion method. It describes the maximum liver (microsomal proteins or hepatocytes) activity towards a compound not affected by other physiological factors such as hepatic blood flow and drug binding within the blood matrix. T${}_{1/2}$ is used to express the time for the 50% disappearance of the parent compound. The apparent intrinsic clearance and T${}_{1/2}$ in hepatocytes for the three TbB ligands are shown in Table 3. A possible explanation for the difference in metabolic stability might be that the TbB ligands bind to microsomes during incubation. This can result in decreased free TbB ligand levels available to interact with metabolizing enzymes, reducing apparent intrinsic clearance values. It is observed that 223-2 is most stable in HLM and most widely metabolized in CPHH.

According to the results, the test compound is extensively depleted in CPHH compared to HLM, thus resulting in significant parent compound depletion. Multiple factors can cause intrinsic clearance by CPHH to differ from HLM [30]. Additional studies such as permeability assay, the impact of the transporter, etc., would be part of our forthcoming studies to gain more insight into the probable reasons for the difference in the intrinsic clearances between HLM and hepatocytes. In summary, the compounds showed varied in vitro half-lives, and intrinsic clearance values, hence, varied metabolic stability. Such a low metabolic stability and high clearance rates by the liver are the two main mechanisms that would be ideally required for the development of potential non-estrogenic NCE.

### 2.6. Single Concentration K${}_{\mathrm{obs}}$ Assay

CYP inhibition evaluation is typically performed by either IC50 at single-point or IC50 Shift or abbreviated assay such as the first-order inactivity rate constant (K${}_{\mathrm{obs}}$) at single concentration. Wong et al. [31] revealed an abbreviated a single K${}_{\mathrm{obs}}$ assay to assess percent inhibition at a single concentration at thirty minutes (30 min) as a single time point, thereby eliminating the requirement for multiple time points necessary for determining the inactivation rate.

This method yielded good correlations between the percent inhibition at 10 or 25 μM and kinact/KI. Evaluation of TD inhibition at single concentration has been implemented in several drug discovery programs [32,33,34]. Evaluation of the single K${}_{\mathrm{obs}}$ approach revealed that, using a database of 400 reference compounds a K${}_{\mathrm{obs}}$ value of 0.02 min${}^{-1}$ (or 45% inhibition after 30 min) is a good indicator of TD inhibition potential [32]. Therefore, in this study, a single K${}_{\mathrm{obs}}$ approach has been utilized to understand the TD inhibition potential of TbB ligands. The results are shown in Table 4. Ideally, the inhibition of CYPs can be reversible or irreversible. The data obtained from single-concentration K${}_{\mathrm{obs}}$ studies were analyzed to understand the same. From the data presented in Table 4, the TbB ligands have values less than 50% for reversible inhibition (R_Inh). As the qualified % reversible inhibition is considered significant if >50%, no significant reversible inhibition is observed for TbB ligands. Knowledge about enzyme inactivation half-life (E½) is also equally important as it would provide insight into the TD inhibition of a compound. E½ can be used to bin the compounds as positive or negative TD inhibitors if the values are <28 or >28, respectively. The TbB ligand 223-2 is observed to be a weak positive TD inhibitor of 1A2 and is negative TD inhibitor to the other six CYP isoforms. On the other hand, the TbB ligand 223-3 is observed to be positive inhibitor of 1A2, 2C9, 2C8, and C19, whereas it is a negative TD inhibitor to the other three CYP isoforms.

**Figure 8:**
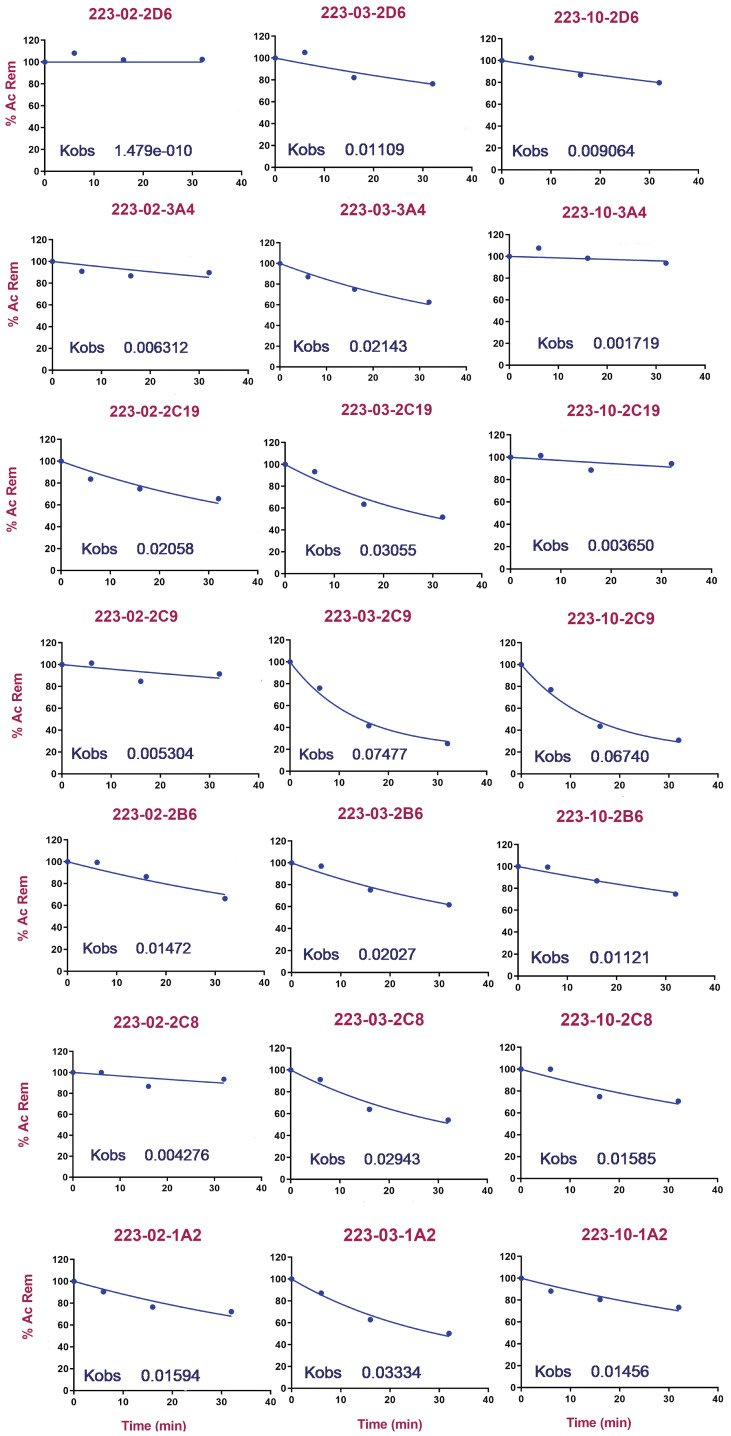
K${}_{\mathrm{obs}}$ values of TbB ligands with different CYPs. A K${}_{\mathrm{obs}}$ value of 0.02 min${}^{-1}$ is a good indicator of TD inhibition potential, and the same is summarized in Table 5.

Similarly, TbB ligand 223-10 is known to positively inhibit 2C9 and 1A2 in a time-dependent manner, whereas it is a negative TD inhibitor to the other CYP isoforms. As previously mentioned, a K${}_{\mathrm{obs}}$ value of 0.02 min${}^{-1}$ is a good indicator of TD inhibition potential; hence such an approach was used to analyze the data as presented in Figure 8. The same is summarized in Table 5 as a traffic light indicator, with green color representing negative inhibition, orange representing weak positive inhibition, and red representing positive inhibition. The TbB ligand 223-2 is observed to be a negative inhibitor of 2C9, 2C8, 2D6, and 3A4 and a weak positive inhibitor of 2B6, 1A2, and 2C19. The TbB ligand 223-3 is observed to be a weak positive inhibitor of 2B6, 2D6, and 3A4 and a positive inhibitor of 2C9, 2C8, 1A2, and 2C19. On the other hand, TbB ligand 223-10 is a negative inhibitor of 2D6, 3A4, and 2C19. It was found to be a weak positive inhibitor of 2B6, 2C8, and 1A2 and a positive inhibitor of 2C9. Therefore, 223-2 is observed to be a weak TD inhibitor when compared to 223-3 and 223-10 TbB ligands.

### 2.7. Bio-Transformation Pathway of TbB Ligands

From the substrate depletion assay, as mentioned in the previous section, it is observed that TbB ligands are undergoing rapid biotransformation in CPHH when compared to HLM. Such a biotransformation might be involving chemical modification of parent ligand by different enzymes, thus generating bio-transformed compounds referred to as metabolites.

Therefore, these metabolites, due to the chemical modifications, might ideally be considered to have increased solubility (hydrophilic form), and considered to be inactive, harmless, and excretable. However, on the contrary, these metabolites can also be formed with higher or equivalent toxicity when compared to the parent ligand. Therefore, to gain such an understanding of metabolite toxicity, 223-2 TbB ligand was shortlisted for metabolite identification (MetID) studies as it was the least prominent CYP inhibitor among the three TbB ligands studied for CYP inhibition, as discussed in earlier sections. The metabolites’ detection and identification were accomplished by utilizing the high-resolution LC-MS/MS (HR LC-MS/MS) technique and the obtained fragmentation pattern was subsequently analyzed to propose putative structures as discussed in the following paragraph. MetID studies was initiated first for the parent 223-2 ligand by HR LC-MS/MS as its fragmentation understanding could act as a guide to potentially identify the metabolites formed after incubation with hepatocytes. Since the 223-2 TbB ligand contain N and O atoms, it could be easily protonated and it was sensitive in positive ionization mode. Consequently, it was studied using the positive fragmentation pattern by ESI-MS${}^{n}$.

**Figure 9:**
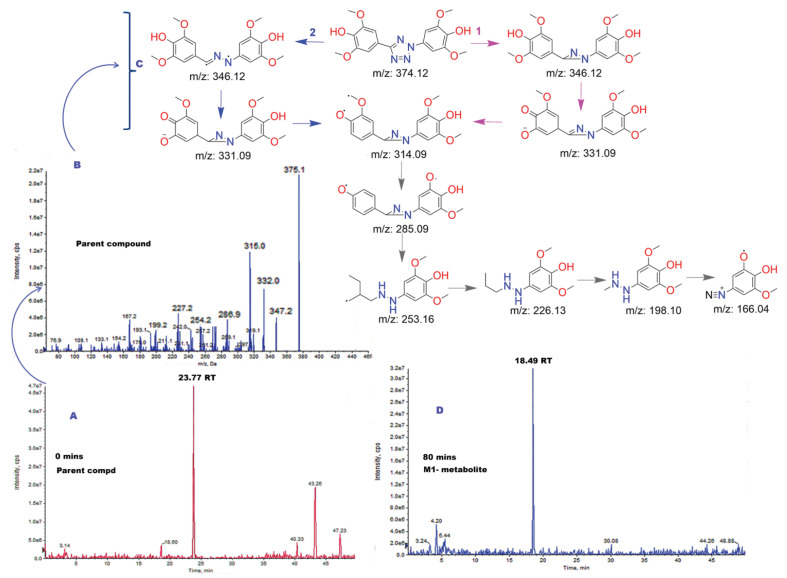
MetID studies of 223-2 incubated in hepatocytes until 80 min. (**A**) Representative chromatogram at 0 min of incubated TbB ligand 223-2 at room temperature. The peak of the parent compound (223-2) is observed at RT of 23.77 min. (**B**) Enhanced product ion (EPI) spectrum with the full range of product ions of 223-2 ligand. (**C**) Schematic representation of the proposed cleavage resulting in the fragmentation of the TbB ligand 223-2 based on the EPI spectra shown in (**B**). (**D**) Representative chromatogram of incubated TbB ligand 223-2 at room temperature highlighting the formation of M1 metabolite at RT of 18.49 min due to metabolism of 223-2 ligand at 80 min.

**Figure 10:**
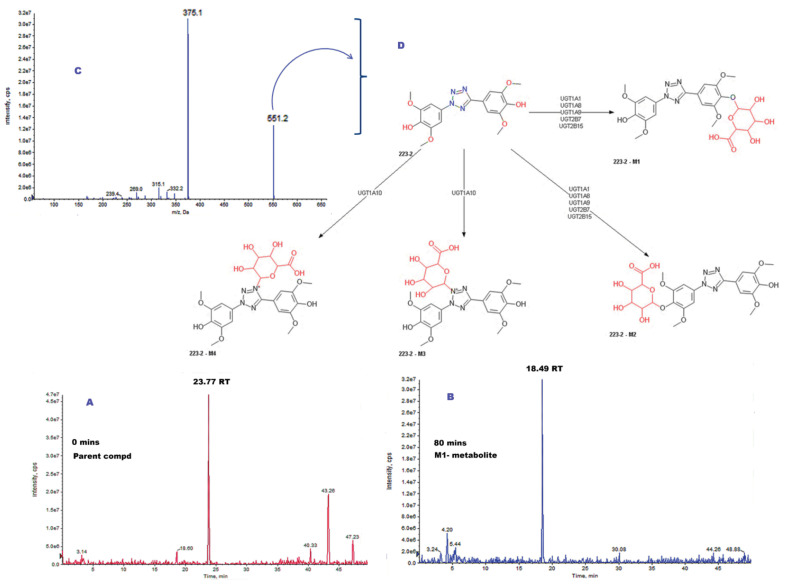
MetID studies of 223-2 incubated in hepatocytes until 80 min. (**A**) Representative chromatogram at 0 min of incubated TbB ligand 223-2 at room temperature. The peak of the parent compound (223-2) is observed at RT of 23.77 min. (**B**) Representative chromatogram of incubated TbB ligand 223-2 at room temperature highlighting the formation of M1 metabolite at RT of 18.49 min due to metabolism of 223-2 ligand at 80 min. (**C**) Enhanced product ion (EPI) spectrum with the full range of M1 metabolite. Based on the fragmentation pattern, the parent ion m/z for this metabolite was found to be 551 (375 + 176). This m/z indicates the formation of glucuronide. (**D**) A schematic representation of the proposed O- and N-glucuronidation of 223-2 ligand, as the exact position of glucuronide binding to compound 223-2 could not be deduced from the fragmentation pattern.

To understand the fragmentation pattern of the parent compound, EPI spectrum was analyzed. Three fragment ions 347.2, 332.0 and 315.0 m/z were considered to propose the possible fragmentation. On closer observation of the ESI-MS${}^{n}$, it is observed that two fragmentation pathways can be proposed, as shown in Figure 9. Both cleavage pathways proceed, likely with the elimination of N2 resulting in either hydrazine or diazirine-based fragment. These fragments undergo further cleavage, resulting in different structures (Figure 9). Such multiple fragmentation pathways can be envisaged due to hetero atoms. Therefore, the initial ionization at specific site determination cannot be precise. Various features like the possibility of resonance stabilization, delocalization throughout the tetrazole ring, and comparable ionization energies of different functional groups could result in the multiple fragmentation pattern. Next, TbB ligand 223-2 was incubated (80 min) with hepatocytes and a new metabolite (M1) could be detected when compared with the control (0 min). The TbB ligand 223-2 was eluted at a retention time of 23.77 min and M1 metabolite at 18.49 min, as seen in Figure 9. Based on the fragmentation pattern, the parent ion m/z for this metabolite was found to be 551 (375 + 176). This m/z indicates the formation of glucuronide (Figure 10). The prominent fragments observed are 375, 315, 332, and 347, which are similar to the fragments observed in TbB ligand 223-2 fragmentation. Although the exact position of glucuronide binding to compound 223-2 could not be deduced from the above fragmentation pattern, based on the in silico metabolites generated from ADMET Predictor, both O- and N-glucuronidation are expected. Formation of such O- and N-glucuronidated metabolites is well known [35]. Glucuronides mostly lack biological activity and are less toxic; for example, BPA-based Glucuronide metabolite was found to be non-estrogenic [6]. Due to limitations in resources and availability of starting materials, the possible O- and N-glucuronide metabolites of TbB ligand 223-2 could not be synthesized for in vitro assay, but in lieu of the same in silico assay, they were performed utilizing the ADMET Predictor to understand their estrogenicity. The results indicated both O- and N-glucuronide metabolites of TbB ligand 223-2 to be non-estrogenic to AR and ER α (Table S3).

The current study provides insight into the metabolism of 2,5-disubstituted tetrazole core-based bisphenol structures. While the scientific consensus indicates that most bisphenol replacements are EDCs whose metabolites have potential toxicity, the current study highlights no potential adverse effect by metabolic activation of polar TbB ligand 223-2. The present study provides a new approach to rethink the existing paradigm for developing safer BPA replacements utilizing the polar heterocyclic core. First, of its instance, our findings highlight the likelihood of tetrazole core-based bisphenol derivatives as privileged compounds for prospective bisphenol replacement. The current study emphasizes opportunities for introducing other heterocycle-based structural motifs into the bisphenol core, thus providing a newer dimension for developing more unique ligands as potential BPA replacement while posing no risk of deleterious metabolic activation effects.

## 3. Materials and Methods

### 3.1. Molecular Docking and Dynamic Simulations

Molecular docking was carried out using seven crystal structures: CYP2C9 (PDB code: 4NZ2), CYP2C8 (PDB code: 2NNI), 2B6 (PDB code: 3IBD), 1A2 (PDB code: 2HI4), 2D6 (PDB code: 4WNV), 3A4 (PDB code: 4D6Z), and 2C19 (PDB code: 4GQ6) after downloading the same from the RCSB data bank [36] (https://www.rcsb.org/, accessed on 21 January 2022 ). Polar hydrogen atoms were added to the protein structure before docking after the water and native ligands were taken out. YASARA molecular modeling software was used to prepare ligands before docking [37]. For each ligand, the conformation with the lowest minimized energy was taken into account during the docking studies. Prior to minimization with default values at physiological pH 7.4 and 0.9% NaCl, ligand solvation was carried out in a solvent box. With default parameters, point charges were first allotted according to the AMBER03 force area field, and then they were damped to emulate the less polar Gasteiger charges used for optimizing the Auto Dock scoring function [38,39]. The macro dock-runlocal.mcr in YASARA was run after each ligand was loaded.

The hydrogen dehydration (HYDE) an implemented scoring function in SeeSAR 12.0 enabled the calculation of the BA post docking [40]. HYDE scoring function depends on the two parameters hydration and desolvation and (atom type-specific). Being an intuitive tool, HYDE enables direct visualization of atom-based scores, thus simplifying the ligand–protein complex analysis to understand the ΔG. An insight into the desolvation, hydrophobicity, and hydrogen bonding effect can be gained by the HYDE scoring function. It enables the understanding of the desolvation penalty as a result of desolvation of hydrophilic atoms, thus contributing unfavorably to the BA. An atom coloring scheme is used in HYDE for intuitive visualization. Three colors—white, red, and green—are utilized, wherein red indicates atoms with unfavourable energy contributions, green indicates atoms that have favourable energetic contributions, and white indicates atoms that have negligible energetic contribution. The estimated BA as the range from mM < μM < nM < pM is visualized. The software SeeSAR from BioSolveIT and Flare from Cresset were utilized for performing molecular docking.

The molecular dynamic simulation of the seven CYP crystal structures was run with YASARA [41]. The setup step involved optimizing hydrogen bonding to maximize the solute stability and finetuning the protonation states of the protein residues at pH 7.4 [42,43]. The cell was neutralized using either excess Na or Cl ions, thus enabling it to maintain 0.9% physiological concentration with NaCl ions. The simulated annealing minimizations with the steepest descent were used to remove clashes. The AMBER14 force field, GAFF2/AMlBCC, and TIP3P were used for the solute, ligands, and water, respectively, for a 50 ns simulation run [44,45,46]. Van der Waals forces in Amber had 8 Å as the default cut-off value, and no limit was set for electrostatic forces using the particle mesh Ewald algorithm [47]. At a temperature of 298 K and a pressure of 1 atm (NPT ensemble), the equations of motion for bonded interactions were integrated with a multiple time step of 1.25 fs (2.5 fs for md-run fast), and 2.5 fs (5.0 fs for md-run fast) for non-bonded interactions using the earlier mentioned algorithm. An MM-PBSA computation using the built-in YASARA binding energy macro was used to evaluate the generated trajectory binding energy.

### 3.2. Source of TbB Ligands

The three 2,5-disubstituted TbB ligands—223-2, 223-3, and 223-10, were available in-house. They were synthesized by 1,5-dipolar cyclization of arylazoaryldiazomethane as they are not commercially available. The synthesized tetrazole derivatives bearing bisphenol structures were analyzed by high-pressure liquid chromatography (HPLC), high-resolution mass spectrometry (HR-MS spectroscopy), ${}^{13}$C NMR, and ${}^{1}$H NMR to confirm their purity and chemical structures. Our previous publication details the synthesis and characterization of these TbB ligands [14].

### 3.3. CYP Inhibition Assay

Using HLM as the enzyme source, the CYP inhibition potential was employed to assess the inhibitory effect of the TbB ligands on the CYP1A2, 2B6, 2C8, 2C9, 2C19, and 2D6 3A4 isoforms. Phenacetin (CYP1A2, 10 mM), tolbutamide (CYP2C9, 20 mM), bupropion (CYP2B6, 10 mM), mephenytoin (CYP2C19, 100 mM), midazolam (CYP3A4, 5 mM), amodiaquine (CYP2C8, 0.02 mM), and dextromethorphan (CYP2D6, 5 mM) were used as probe substrates. The test substance included TbB ligand (final concentrations of 10 M for each) and HLMs (final concentration of 0.1 mg protein per mL), with or without 1.0 mM NADPH. The reaction was started by adding pre-incubated 25 μL of NADPH into all wells and incubate at 37 °C for 10 min for 3A4 and 20 min for remaining all isoforms. The reaction was stopped by adding 100 μL of stop solution to all wells. The samples were centrifuged at 4000 rpm for 10 min at 4 °C. Supernatant (100 μL) was withdrawn, mixed with 200 μL of water followed by LC-MS/MS analysis.

### 3.4. Substrate Depletion Assay

By preincubating a test substance for 5 minutes in phosphate buffer (pH 7.4) at 37 °C in shaking water bath, the stability of the test substance was examined in pooled HLM. After NADPH-generating systems addition, the reaction gets initiated and post incubation for 0, 15, 30, 45, and 60 min, the reaction is terminated with addition of acetonitrile/methanol to the incubation mixture. Supernatant obtained after mixing and centrifuging the sample is analyzed by HPLC-MS/MS. Microsomal protein for final use is at 0.1 mg/mL concentration. Four compounds, namely, propranolol and imipramine, known to be relatively stable, and verapamil and terfenadine, known as readily metabolizing in HLM, were included in each assay. Post HPLC-MS/MS of the samples, data are analyzed by considering test compounds peak area at each time point to time zero. Assuming the reaction to follow first-order kinetics, the slope of the initial linear range of the logarithmic curve of remaining compound (%) vs. time is considered to calculate the half-life. Additionally, the half-life is utilized to calculate the intrinsic clearance (CLint) as shown in the following equation.

$$\mathrm{CL}{}_{int}(\mu L/\min/\mathrm{mg}\;\mathrm{protein})\;=\;0.693/T{}_{1/2}\times \mathrm{protein}\;\mathrm{conc}.$$

The test compound stability in a pooled human cryopreserved hepatocytes was tested after thawing, washing, and resuspending the cryopreserved hepatocytes in Krebs–Heinslet buffer (pH 7.3). The onset of reaction happens by the addition of the test compound into cell suspension and incubated for 0, 30, 1, 1.5, and 2 h, respectively, at 37 °C/5% CO${}_{2}$. The reaction is terminated in the incubation mixture by the addition of acetonitrile. After thorough sample mixing, the sample was completely transferred to another 96-well plate and centrifuged, followed by HPLC-MS/MS analysis of supernatant. Four compounds as reference, namely, propranolol, a relatively stable and readily metabolizing flurazepam, naloxone, and HFC (7-hydroxy-4-trifluoromethyl-coumarin) in human hepatocytes were included in each assay. Data analysis was performed as mentioned above and the intrinsic clearance (CLint) is calculated from the half-life using the following equation.

$$\mathrm{CL}{}_{int}(\mu L/\min/\;\mathrm{million}\;\mathrm{cells})\;=\;0.693/T{}_{1/2}\times \mathrm{Cell}\;\mathrm{Density}.$$

### 3.5. Single Concentration K${}_{\mathrm{obs}}$ Assay

The time-dependent inhibition (TDI) potential of test compounds against CYP1A2, 2B6, 2C8, 2C9, 2C19, 2D6, 3A4/5 isoforms were determined using Human liver microsomes. Assay is performed in 96-deep well plate format (n = 1). Positive controls and DMSO as negative control in each assay. To obtain 100 μM working stock solution, 1 μL of blank DMSO/test/reference compounds to 100 μL 1× DPBS buffer was added and labeled as dilution plate. To the primary incubation plate, 45 μL of primary mix was dispensed to the desired wells containing 25 μL HLM, 10 μL MgCl2, and 10 μL of NADPH working solutions. To the secondary incubation plate, 95 μL of secondary mix was dispensed to the desired wells containing 20 μL MgCl2, 20 μL NADPH, 10 μL respective substrates for CYP isoform working solutions and 45 μL 1× DPBS buffer. Each of these four plates were labeled with their respective time points, i.e., 0, 6, 16, 32 min. Pre-incubated the dilution plate along with primary incubation plate at 37 °C for 15 min at 450 rpm. The reaction was carried out in triplicate. After the incubation, the reaction was quenched by adding 150 μL ice cold stop solution to all the wells followed by centrifuging the samples at 4000 rpm, 4 °C for 15 min. The supernatant samples were then transferred into vials for LC-MS/MS analysis. The percent Reversible Inhibition was calculated using the following formula.

$$\%\mathrm{Reversible}\mathrm{inhibition}=100-(\mathrm{PAR}\mathrm{test}\mathrm{comp}t_{0}\times 100)/(\mathrm{PAR}\;\mathrm{average}\;\mathrm{DMSO}\;t{}_{0})$$

### 3.6. LC-MS/MS Method for Metabolite Identification

A liquid chromatography–high resolution mass spectrometer (LC-MS/MS 5500 + QTRAP) was utilized for the metabolite’s identification formed during the MetID studies. A gradient elution using the mobile phase A (containing water and 0.1% of formic acid) and mobile phase B (containing acetonitrile and 0.1% of formic acid) was used for separation on Phenomenex, Luna C18, 100 A, 5 μm. Gradient elution was performed with 5% B for 2 min, then ramped up to 95% in 36 min, holding at 95% for 10 min, and returned to initial conditions over 1 min. Four minutes of equilibration time followed, resulting in a total run time of 50 min. The Applied Biosystems MDS SCIEX, 5500 QtrapMS/MS was equipped with a heated electrospray ionization source along with positive ionization mode. The spray voltage used was 5500 V, ion source temperature was set to 550 C with GS1 (Nebuliser gas) and GS2 (Auxiliary gas) at 55 psi and 60 psi, respectively. The collision gas along with CUR (Curtain gas), set at 10 psig and 30 psig, respectively, were adjusted to give maximum sensitivity. The MS was run in Multiple Reaction Monitoring (MRM) mode along with positive polarity EPI scanning using Information Dependent Acquisition (IDA), enabling utilization of MRM ratios and MS/MS spectra for compound identification.

## 4. Conclusions

This study investigated the in silico and in vitro metabolism of 2,5-disubstituted tetrazole derivatives-based bisphenols (TbB). The CYP inhibition assay results in a single concentration of 10 μM show that 223-3 ligand was observed to have above 50% inhibitory effect on the CYP isoforms CYP2C8, CYP2D6, CYP2C9, CYP2B6, CYP3A4, and CYP2C19. The 223-10 ligand had inhibition potential for CYP2B6 and CYP2C8, whereas 223-2 inhibited only CYP2C9. None of the three ligands inhibited CYP1A2. The ligand 223-3 was found to be a potential time-dependent inhibitor for 2C9, 2C19, and 1A2 with K${}_{\mathrm{obs}}$ values of 0.0748, 0.0306, and 0.0333 min${}^{-1}$, respectively. On the other hand, the TbB ligand 223-10 was found to inhibit 2C9 in a time-dependent manner with K${}_{\mathrm{obs}}$ value 0.0748, min${}^{-1}$, whereas 223-2 showed a weak TD inhibition effect on 2C19, CYP1A2, and CYP2B6 with K${}_{\mathrm{obs}}$ values of 0.0206, 0.0159, and 0.0147 min${}^{-1}$, respectively. Therefore, TbB ligands 223-3 and 223-10 were observed to be potential CYP inhibitors with TD inhibition potential, whereas the TbB 223-2 ligand did not show such a significant effect. It is observed that the TbB ligand 223-3 is metabolized by the phase 1 enzymes of HLM whereas the other TbB ligands demonstrated stability in the HLM. In contrast, all three TbB ligands were metabolized in cryopreserved hepatocytes with only <20% of the parent compound remaining, suggesting that these ligands predominantly undergo phase II metabolism or with non-CYP pathways. The 223-2 ligand metabolites biotransformation pathway suggested the clearance via glucuronidation and highlighted no potential adverse effect by metabolic activation. The overall results of current study highlight 223-2 TbB ligand as privileged BPA replacement, however, the results strongly warrant further in vivo DDI and MetID studies.

## Figures and Tables

**Table 1:** Molecular interactions of 223-2 with active site residues of representative CYPs.

	Molecular Interactions of 223-2		
**Ligand-CYP Complex**	**% Hydrophobic Interactions**	**% Hydrophobic + H-Bond Interactions**	**% H-Bond Interactions**
223-2-1A2	18 residues, avg. contact/residue 58%	37%	4% from Thr 321
223-2-2B6	19 residues, avg. contact/residue 52%	-	31% from Lys 479

**Table 2:** Molecular interactions of 223-3 with active site residues of representative CYPs.

	Molecular Interactions of 223-3		
**Ligand-CYP Complex**	**% Hydrophobic Interactions**	**% Hydrophobic + H-Bond Interactions**	**% H-Bond Interactions**
223-3-2D6	20 residues, avg. contact/residue 45%	-	84% from Ser 304
223-3-3A4	16 residues, avg. contact/residue 45%	35%	37% from Glu 374

**Table 3:** Intrinsic clearance studies with CPHH at test conc. of $1\times 10^{-6}$ M (n = 3), SD = standard deviation.

Ligands	Half-Life (Min)		CLint (μL/min/million)
	Mean	SD	
223-2	33	1.0	29.8
223-3	54	2.3	18.4
223-10	36	4.4	27.8

**Table 4:** Results of TbB ligands by a single K${}_{\mathrm{obs}}$ assay to assess percent inhibition at a single concentration at thirty minutes (30 min) as single time point. This approach has been utilized to understand the TD inhibition potential of the TbB ligands. The qualified % Reversible Inhibition (R_Inh) %) is considered significant if >50%. The enzyme inactivation half-life (E½) is used to bin the compounds as positive or negative TD inhibitors if the values are <28 or >28, respectively.

Ligands	**2C9**		**2B6**		**2C8**		**1A2**	
	**R_Inh%**	**E½**	**R_Inh%**	**E½**	**R_Inh%**	**E½**	**R_Inh%**	**E½**
223-2	<50	>28	<50	>28	<50	>28	<50	21.54
223-3	<50	9.27	<50	>28	<50	23.55	<50	18.38
223-10	<50	10.28	<50	>28	<50	>28	<50	20.25
Ligands	**2D6**		**3A4**		**2C19**			
	**R_Inh%**	**E½**	**R_Inh%**	**E½**	**R_Inh%**	**E½**		
223-2	<50	>28	<50	>28	<50	>28		
223-3	<50	>28	<50	>28	<50	22.69		
223-10	<50	>28	<50	>28	<50	>28		

**Table 5:** Single-concentration K${}_{\mathrm{obs}}$ assay of TbB ligands. A K${}_{\mathrm{obs}}$ value of 0.02 min${}^{-1}$ is a good indicator of TDI potential. The same is presented in the table as a traffic light indicator, with green color representing negative inhibition, orange representing weak positive inhibition, and red representing positive inhibition. Based on the single concentration K${}_{\mathrm{obs}}$ assay, the TD inhibition potential of the ligands can be expressed as 223-3 > 223-10 > 223-2. Therefore, 223-2 is a weak TD inhibitor.

	Single Concentration K${}_{\mathrm{obs}}$ Assay (Risk Bin)						
**Ligand**	**2C9**	**2B6**	**2C8**	**1A2**	**2D6**	**3A4**	**2C19**
223-2	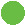	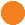	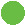	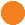	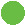	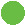	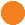
223-3	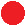	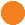	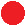	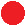	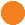	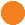	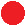
223-10	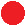	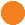	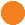	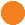	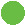	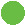	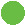

## Data Availability

The manuscript’s supporting information section provides the supporting data. Readers can download the crystal structures of CYP2C9 (PDB code: 4NZ2), CYP2C8 (PDB code: 2NNI), 2B6 (PDB code: 3IBD), 1A2 (PDB code: 2HI4), 2D6 (PDB code: 4WNV), 3A4 (PDB code: 4D6Z), and 2C19 (PDB code: 4GQ6) used in the study from the RCSB data bank (https://www.rcsb.org/, accessed on 21 January 2022). The structure-based design, ligand-based design, molecular dynamic simulations, and in silico assay performed in the study utilized the following licensed software: (1) Flare (http://www.cresset-group.com/flare/, accessed on 18 March 2022); (2) SeeSAR (https://www.biosolveit.de/download/, accessed on 15 April 2022); (3) YASARA (http://www.yasara.org/, accessed on 21 January 2022); (4) ADMET predictor (https://www.simulations-plus.com/software/admetpredictor/, accessed on 18 March 2022). The respective developers can be reached for evaluation licenses considering the propriety aspects of the software. Readers can request a free trial of the statistical analysis tool Prism, used in the current study, (https://www.graphpad.com/scientific-software/prism/ accessed on 30 January 2023 ). ADMET of the studied ligands can be estimated using the Optibrium and StarDrop model runner algorithms in SeeSAR v.10.3 (https://www.biosolveit.de/download/ accessed on 15 April 2022). All the other data are available upon request.

## Supplementary Materials

The following supporting information can be downloaded at: https://www.mdpi.com/article/10.3390/molecules28031465/s1, Table S1: HYDE Binding affinity comparison of ligands obtained using SeeSAR for the seven CYP isoforms, Figure S1: Docked poses of TbB ligands in the active site of CYP2C9, Figure S2: Docked poses of TbB ligands in the active site of CYP2C8, Figure S3: Docked poses of TbB ligands in the active site of CYP2D6, Figure S4: Docked poses of TbB ligands in the active site of CYP3A4, Figure S5: Docked poses of TbB ligands in the active site of CYP2C19, Figure S6: DFT optimized chemical structure of the ligands highlighted with the energy gaps, Table S2: Molecular properties of the ligands, Figure S7: Prediction of SOM of 223-2 ligand by Xenosite server, Figure S8: Prediction of SOM of 223-3 ligand by Xenosite server, Figure S9: Prediction of SOM of 223-10 ligand by Xenosite server, Figure S10: Predicted sites of reactivity (SOR) for the three TbB ligands by online XenoSite reactivity model, Figure S11: Predicted biotransformation of 223-10 ligand by phase-1 and phase 2 pathway, Figure S12: Predicted biotransformation of 223-3 ligand by phase-1 and phase 2 pathway, Figure S13: Predicted biotransformation of 223-10 ligand by phase-1 and phase 2 pathway, Table S3: In silico assay of TbB ligands metabolites generated by phase 1 enzymes and phase II enzymes, Figures S14–S17: The TbB ligands movement RMSD after MD simulations in each of CYPs, Figure S18: Metabolic stability studies of TbB ligands in HLM, Table S4: Intrinsic clearance comparative studies with HLM and CPHH at test conc. 1.0 × 10^−7^ M and 1.0 × 10^−6^ M respectively.

## Sample Availability

Samples of the compounds are not available from the authors.

## Abbreviations

The following abbreviations are used in this manuscript:

BPA	bisphenol acetone
TbB	tetrazole derivatives bearing bisphenol
NCE	new chemical entity
MBP	4-methyl-2,4-bis(p-hydroxyphenyl) pent-1-ene
SOM	site of metabolism
ADMET	absorption, distribution, metabolism, excretion, and toxicity
HLM	human liver microsome
CPHH	cryopreserved human hepatocytes
TDI	time-dependent inhibitor/inhibition
DDI	drug–drug interaction
BA	binding affinity
GSH	glutathione; NADPH, nicotinamide adenine dinucleotide phosphate
UDPGA	uridine diphosphate glucuronic acid
UGTs	UDP-glucuronosyltransferase
ESI-MSn	electrospray ionization multistage mass spectrometry
U.S.EPA	U.S. Environmental Protection Agency
RBA	relative binding affinity
LBP	ligand binding pocket
SAR	structure–activity relationship
FDA	Food and Drug Administration
RMSD	root-mean-square deviation
HR-MS	high-resolution mass spectroscopy
HYDE	Hydrogen bond and Dehydration energies
PAR	percent remaining activity of enzyme
