# Supplementary material for: An Insight into the Metabolism of 2,5-Disubstituted Monotetrazole Bearing Bisphenol Structures: Emerging Bisphenol A Structural Congeners
Source: Molecules. 2023 Feb 2;28(3):1465. doi: 10.3390/molecules28031465 (PMC9921896; doi:10.3390/molecules28031465)
Supplement: Supplementary file 1 [file molecules-28-01465-s001.zip › molecules-2123612 supplementary materials.pdf]

molecules

Supplementary Materials

An Insight into the Metabolism of 2,5-Disubstituted Monotetrazole Bearing Bisphenol Structures: An Emerging Bisphenol A Structural Congeners

Umesh B. Gadgoli [1,*,†] Yelekere C. Sunil Kumar [2,†] and Deepak Kumar [1,†]

1. Results and Discussion

1.1. Hydrogen Bond and Dehydration Energy (HYDE)

Table S1. HYDE Binding affinity comparison of ligands obtained using SeeSAR for the seven CYP isoforms.

Ligand	log P	HYDE BA (KJ/mol) 2C9-	2B6-	2C8-	1A2-	2D6-	3A4-	2C19-
		4NZ2	3IBD	2NNI	2HI4	4WNV	4D6Z	4GQS
BPA	3.42	-30.3	-41	-24.2	-41.3	-27	-12	-36.3
223-2	1.77	-17.8	-14.8	-24.4	-	-4.7	-9.6	-19.7
223-3	2.97	-21.9	-	-19	-	-27.3	-25	-33.4
223-10	1.74	-21.8	-11.3	-24.2	-29.4	-16.5	-12.8	-12.1

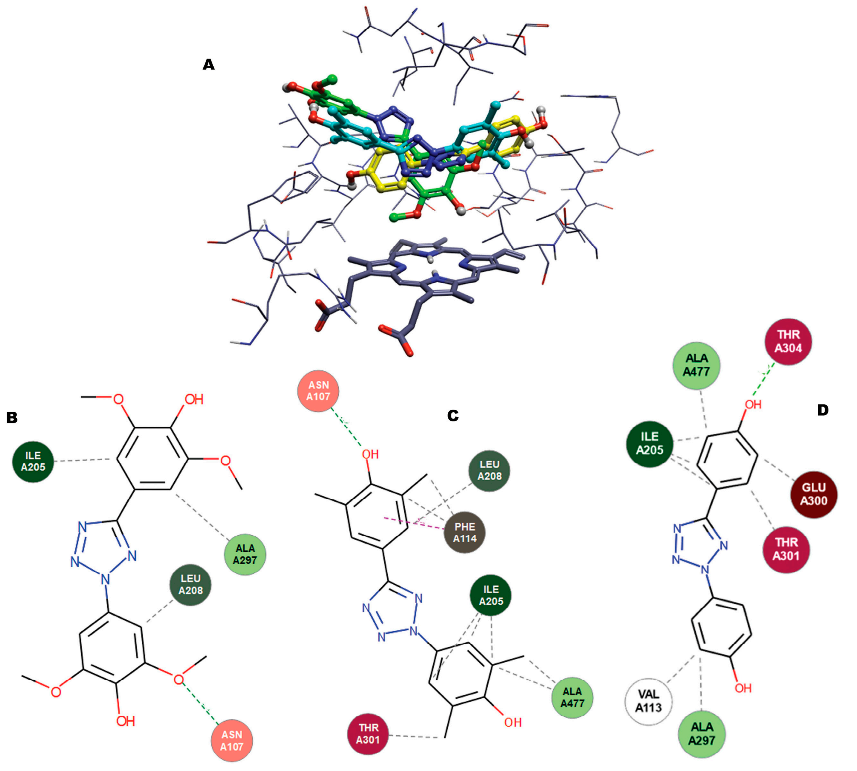

Figure S1. CYP2C9 (A) Docked poses of TbB ligands in the active site of CYP2C9. Green pose = 223-2. Cyan pose = 223-3. Yellow pose = 223-10. Heme group = light rose. (B) Molecular interactions of 223-2 with the active site residues. (C) Molecular interactions of 223-3 with the active site residues. (D) Molecular interactions of 223-10 with the active site residues. Molecular interactions represented as dotted lines are hydrogen bonds, hydrophobic connects and aromatic-aromatic interactions.

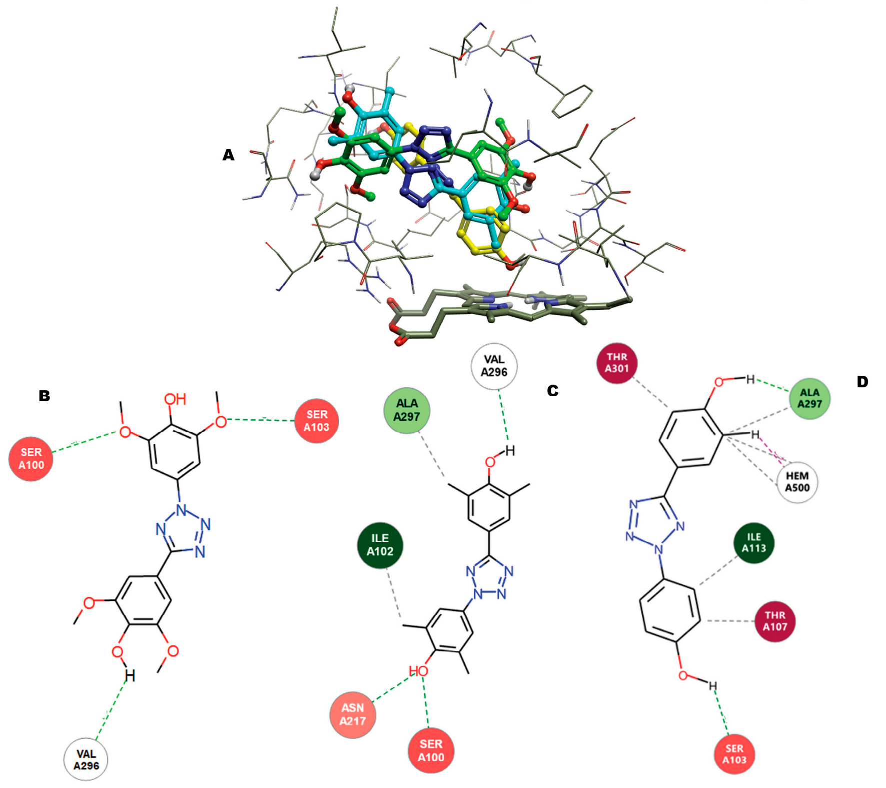

Figure S2. CYP2C8: (A) Docked poses of TbB ligands in the active site of CYP2C8. Green pose = 223-2. Cyan pose = 223-3. Yellow pose = 223-10. Heme group = light rose. (B) Molecular interactions of 223-2 with the active site residues. (C) Molecular interactions of 223-3 with the active site residues. (D) Molecular interactions of 223-10 with the active site residues. Molecular interactions represented as dotted lines are hydrogen bonds, hydrophobic connects and aromatic-aromatic interactions.

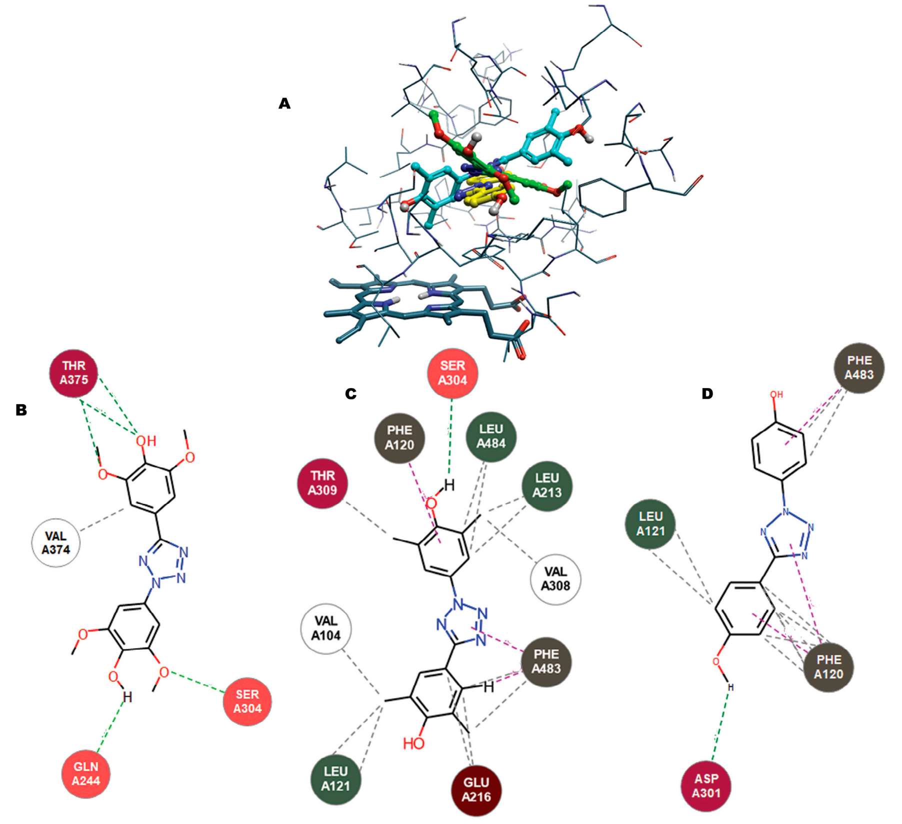

Figure S3. CYP2D6 : (A) Docked poses of TbB ligands in the active site of CYP2D6. Green pose = 223-2. Cyan pose = 223-3. Yellow pose = 223-10. Heme group = light rose. (B) Molecular interactions of 223-2 with the active site residues. (C) Molecular interactions of 223-3 with the active site residues. (D) Molecular interactions of 223-10 with the active site residues. Molecular interactions represented as dotted lines are hydrogen bonds, hydrophobic connects and aromatic-aromatic interactions.

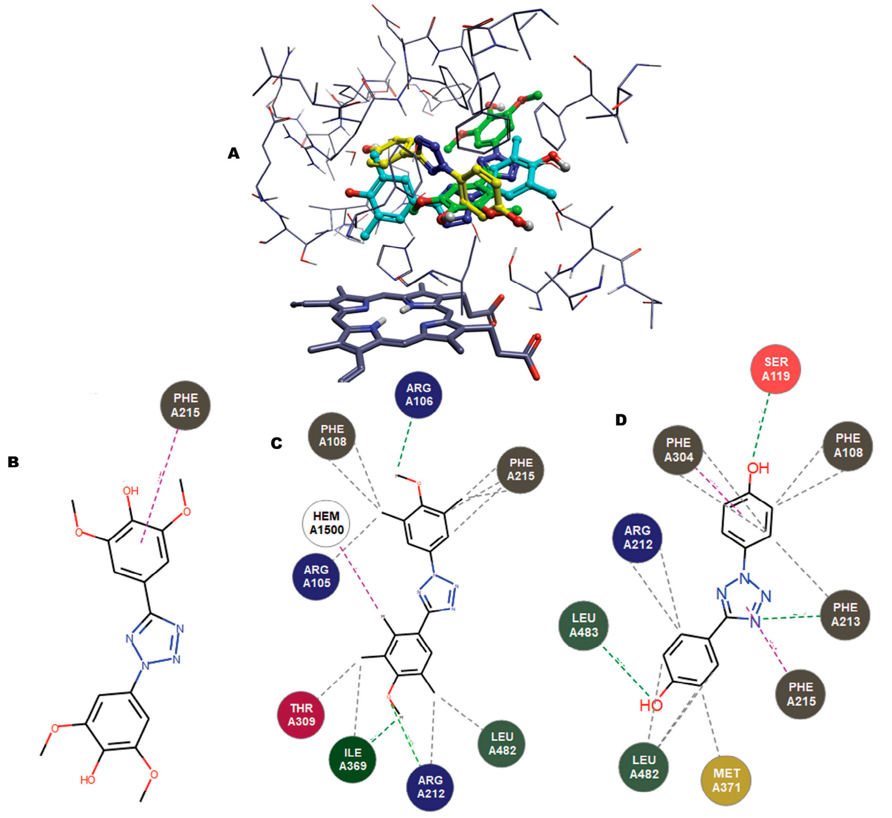

Figure S4. CYP3A4 : (A) Docked poses of TbB ligands in the active site of CYP3A4. Green pose = 223-2. Cyan pose = 223-3. Yellow pose = 223-10. Heme group = light rose. (B) Molecular interactions of 223-2 with the active site residues. (C) Molecular interactions of 223-3 with the active site residues. (D) Molecular interactions of 223-10 with the active site residues. Molecular interactions represented as dotted lines are hydrogen bonds, hydrophobic connects and aromatic-aromatic interactions.

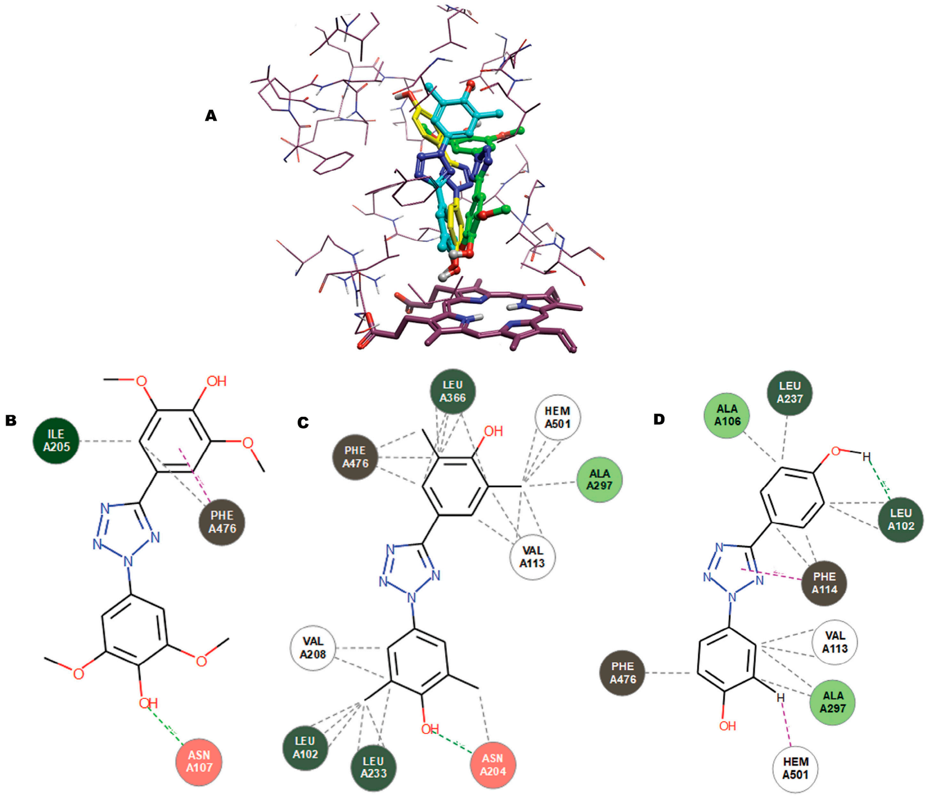

Figure S5. CYP2C19 : (A) Docked poses of TbB ligands in the active site of CYP2C19. Green pose = 223-2. Cyan pose = 223-3. Yellow pose = 223-10. Heme group = light rose. (B) Molecular interactions of 223-2 with the active site residues. (C) Molecular interactions of 223-3 with the active site residues. (D) Molecular interactions of 223-10 with the active site residues. Molecular interactions represented as dotted lines are hydrogen bonds, hydrophobic connects and aromatic-aromatic interactions.

1.2. Density Function Theory (DFT) Calculations

The intermolecular charge and electronic transitions calculations using DFT are known in medicinal research to explicate the correlation between structural features and drug molecule binding properties. DFT furnishes the compounds frontier molecular orbitals, highest occupied molecular orbital (HOMO), and lowest unoccupied molecular orbital (LUMO), which determines an organic compound's chemical stability. HOMO illustrates the electron donor, and LUMO illustrates the electron acceptor capability. The energy gap between the HOMO-LUMO refers to the potential energy difference. It indicates the amount of energy needed for the excitation from the stable state (ground state) to the excited state. Therefore, the energy gap signifies a molecule's chemical reactivity. The DFT calculated energies associated with HOMO-LUMO is summarized in Table 3. The data presented in Table 3 shows that the BPA has a higher energy gap than the tetrazole-based bisphenol. Therefore, it has comparatively increased chemical hardness than the TbB ligands. In other words, TbB ligands are softer and require less energy to get activated; therefore, they have a higher ability to polarize than hard molecules like BPA. Therefore, the TbB ligands, because of their higher reactivity, might be unstable. On the other hand, a dipole moment typically influences the magnitude of interactions, for example, hydrogen bond formation and non-covalent interaction. Hence, the higher the dipole moment, the better the binding ability. It could, therefore, be predicted that 223-2 and 223-3 TbB ligands

might bind better

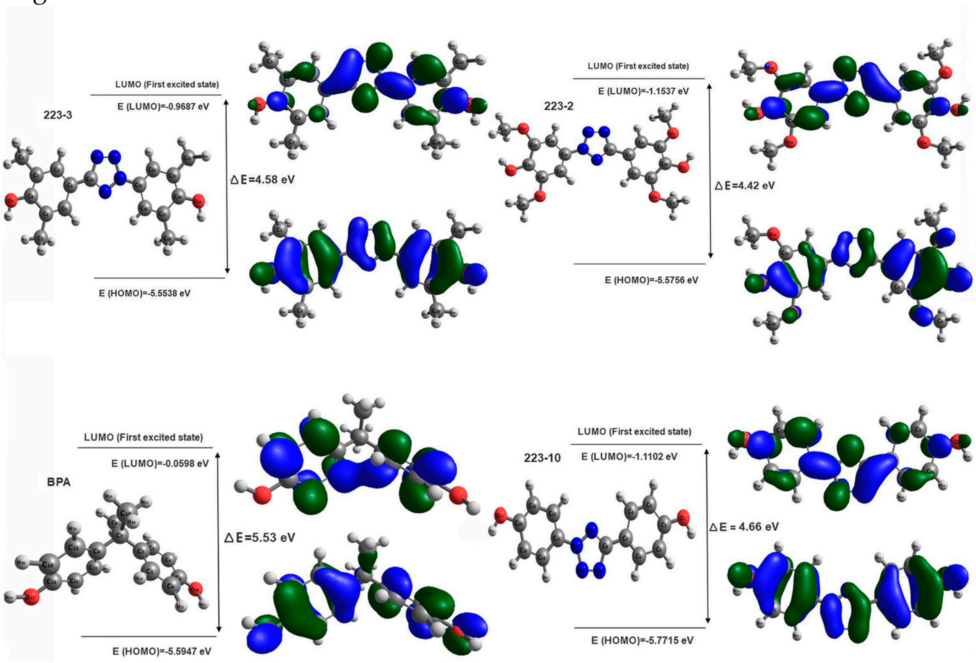

Figure S6. DFT optimized chemical structure of the ligands highlighted with the energy gaps.

Table S2. Molecular properties of the ligands.

Ligands	HOMO (A.U)	LUMO (A.U)	Gap (A.U)	Dipole Moment (Debye)	Hardness (eV)	Softness (eV-1)	Chemical potential (eV)	Electron Affinity (eV)
BPA	-0.2224	-0.0012	-0.2212	2.57	0.1106	4.5207	-0.1118	0.0565
223-2	-0.1815	-0.005	-0.1765	5.666	0.0883	5.6654	-0.0933	0.0493
223-3	-0.1903	-0.0078	-0.1825	5.642	0.0913	5.4793	-0.099	0.0537
223-10	-0.1953	-0.007	-0.1883	1.458	0.0941	5.3117	-0.1011	0.0543

Due to higher dipole moment. However, 223-10, which has less dipole than BPA, might be less bound than BPA. Although the in silico predictions provided an understanding of the binding affinity, SOM, CYP inhibition, and molecular properties of TbB ligands, such modeling results alone are insufficient for verifying the finding considering the limitations, hence in vitro assay's were performed to corroborate the in silico predictions. The molecular docking and HYDE prediction provided insightful information on how avidly the ligands bind to a given P450 enzyme. Molecular docking suggested that in some of the CYP, the atoms of some poses are within the 6 Å of the heme iron; however, to corroborate prediction of the metabolism site (SOM), which is key to understanding the ligand's metabolic stability or instability, the additional computational tools were utilized such as the ADMET Predictor Metabolism module and XenoSite Metabolism and Reactivity Prediction Web Server.

1.3. Sites of Metabolism

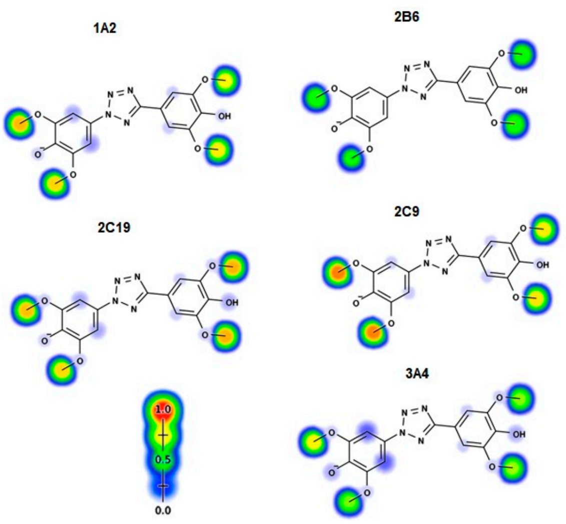

Figure S7. Prediction of SOM of 223-2 ligand by Xenosite server, labeling sites of metabolism for different CPY isoforms. XenoSite provides visual output wherein a color gradient labels the potential SOMs. zero probability of metabolism is represented by blue, a probability equal to the background probability of observing a SOM at random is represented by white, and a probability of 1.0 is indicated by red.

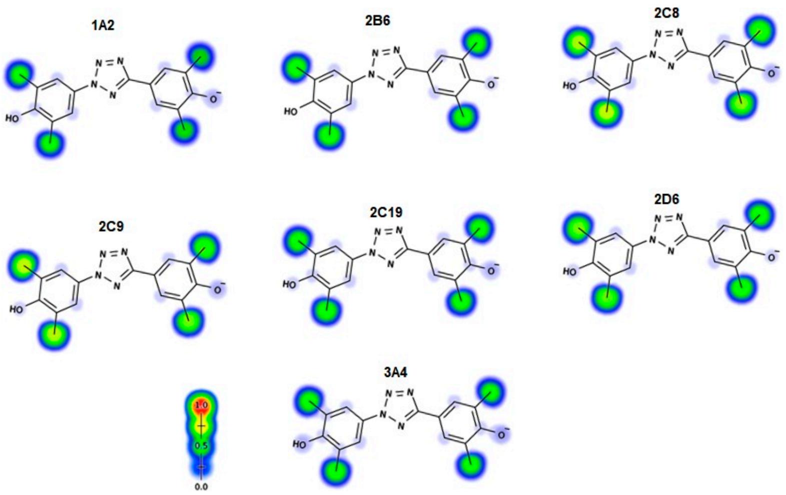

Figure S8. Prediction of SOM of 223-3 ligand by Xenosite server, labeling sites of metabolism for different CPY isoforms. XenoSite provides visual output wherein a color gradient labels the potential SOMs. zero probability of metabolism is represented by blue, a probability equal to the background probability of observing a SOM at random is represented by white, and a probability of 1.0 is indicated by red.

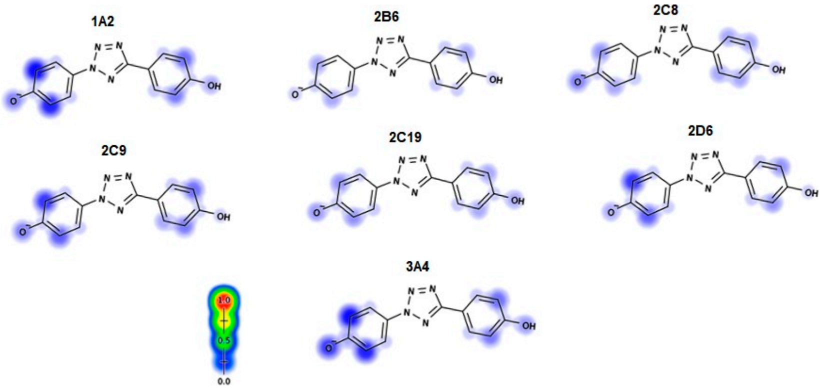

Figure S9. Prediction of SOM of 223-10 ligand by Xenosite server, labeling sites of metabolism for different CPY isoforms. XenoSite provides visual output wherein a color gradient labels the potential SOMs. zero probability of metabolism is represented by blue, a probability equal to the background probability of observing a SOM at random is represented by white, and a probability of 1.0 is indicated by red.

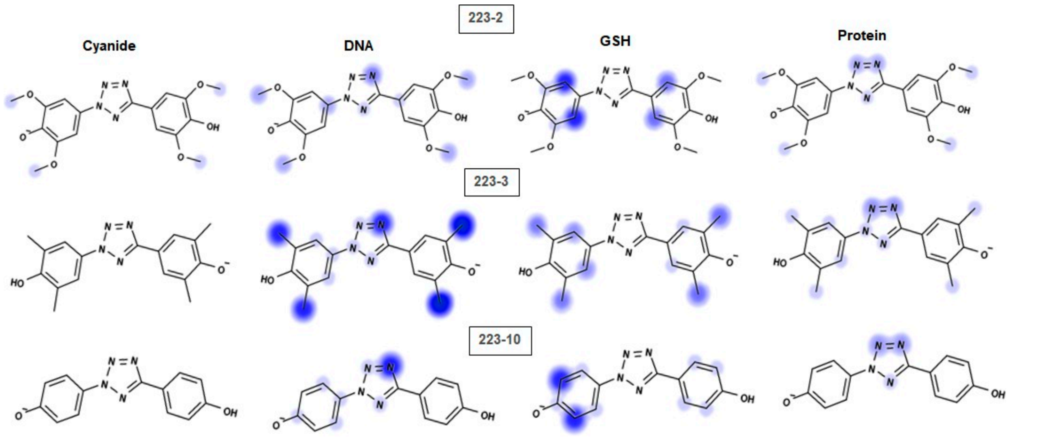

Figure S10. Predicted sites of reactivity (SOR) for the three TbB ligands by online XenoSite reactivity model. Model enabled to determine the labile electrophilic sites on atoms of TbB ligands susceptible to DNA, cyanide, GSH, and protein.

1.4. In Silico Predicted Bio-Transformations

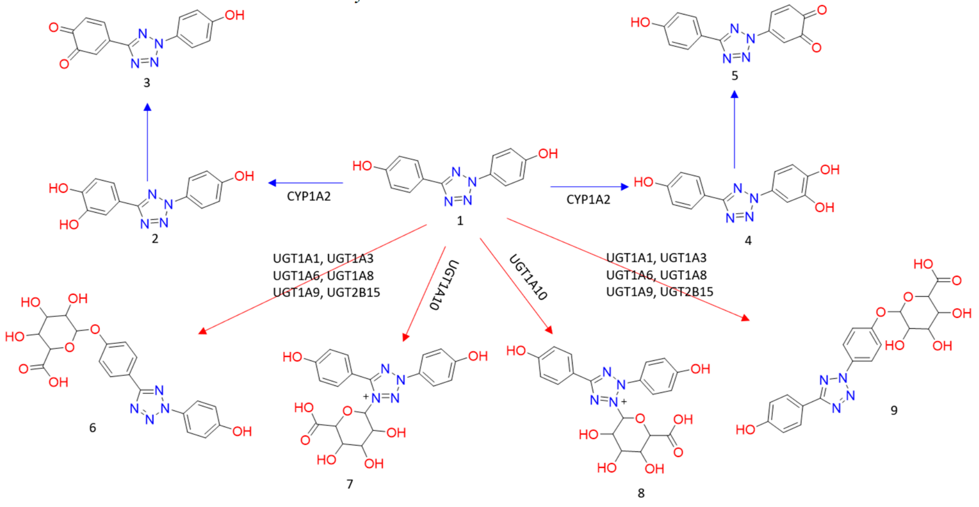

Figure S11. Predicted bio-transformation of 223-10 ligand by phase-1 and phase-2 pathway.

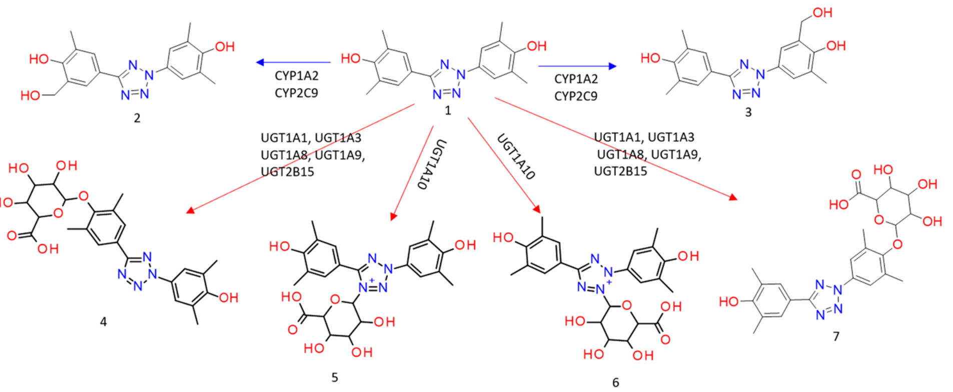

Figure S12. Predicted bio-transformation of 223-3 ligand by phase-1 and phase-2 pathway.

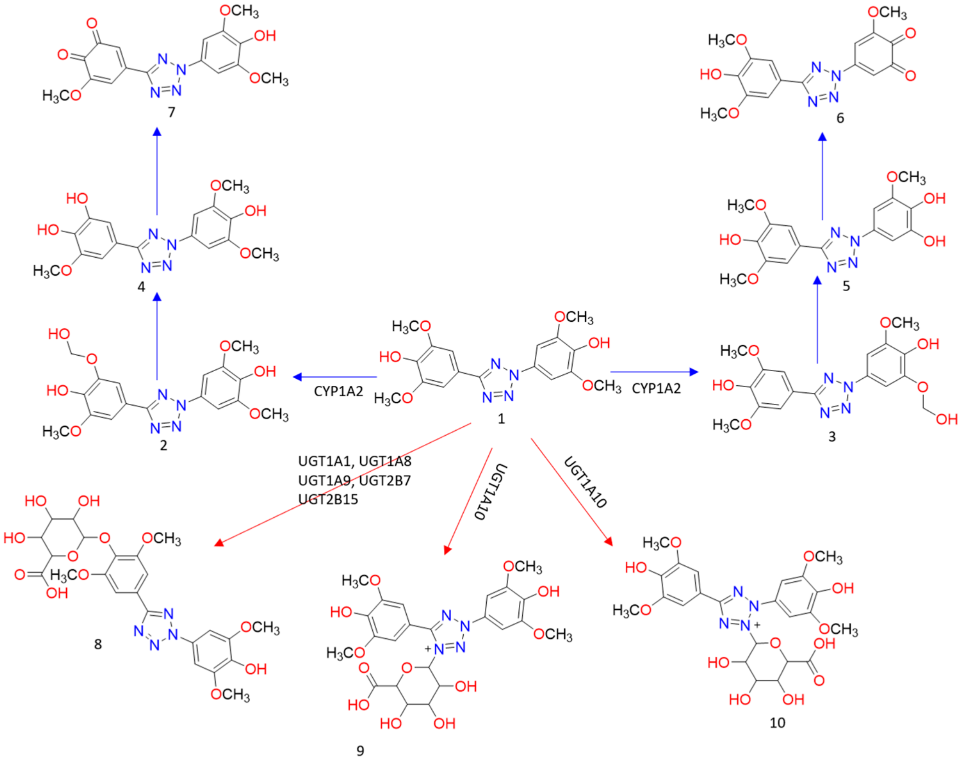

Figure S13. Predicted bio-transformation of 223-2 ligand by phase-1 and phase-2 pathway.

Table S3. In silico assay of TbB ligands metabolites generated by phase 1 enzymes and phase II enzymes. ADMET predictor qualitatively assesses the estrogen (estro) and androgen (andro) receptor toxicity in rats. The output is shown as Toxic or Nontoxic. If the ligand is found Toxic then it is considered as ER or AR active and if the ligand is found Nontoxic, then it is considered as ER or AR inactive. The underlined value highlights that data is not reliable as it might not be in the applicability domain..

Name	Estro filter	Estro RBA	Andro filter	Andro RBA
223-10 (1)	Nontoxic (49%)	Nontoxic	Toxic (89%)	0.011
223-10 (2)	Toxic (63%)	0.037	Nontoxic (48%)	Nontoxic
223-10 (3)	Nontoxic (85%)	Nontoxic	Nontoxic (77%)	Nontoxic
223-10 (4)	Toxic (63%)	0.038	Toxic (48%)	0.004
223-10 (5)	Nontoxic (49%)	Nontoxic	Nontoxic (80%)	Nontoxic
223-10 (6)	Nontoxic (85%)	Nontoxic	Toxic (61%)	0.002
223-10 (7)	Nontoxic (85%)	Nontoxic	Toxic (59%)	0.002
223-10 (8)	Toxic (56%)	9.603E-5	Toxic	0.005
223-10 (9)	Toxic	1.007E-5	Toxic (84%)	0.017

223-3 (2)	Nontoxic (51%)	Nontoxic	Toxic (66%)	0.016
223-3 (3)	Nontoxic (51%)	Nontoxic	Toxic (66%)	0.016
223-3 (4)	0.025	Toxic (57%)	Nontoxic	Nontoxic (85%)
223-3 (5)	Toxic (76%)	0.210	Toxic	1.423E-4
223-3 (6)	Toxic	0.070	Toxic 59%)	1.343E-4
223-3 (7)	0.024	Toxic (57%)	Nontoxic	Nontoxic (85%)
223-2 (4)	Nontoxic (98%)	Nontoxic	Toxic (68%)	0.073
223-2 (5)	Nontoxic (98%)	Nontoxic	Toxic (68%)	0.072
223-2 (6)	Nontoxic	Nontoxic	Toxic (63%)	0.014
223-2 (7)	Nontoxic	Nontoxic	Toxic (49%)	0.022
223-2 (8)	Nontoxic (87%)	Nontoxic	Nontoxic (79%)	Nontoxic
223-2 (9)	Nontoxic	Nontoxic	Toxic (63%)	1.951E-4
223-2 (10)	Nontoxic (77%)	Nontoxic	Toxic	2.280E-4

1.5. MD Simulations

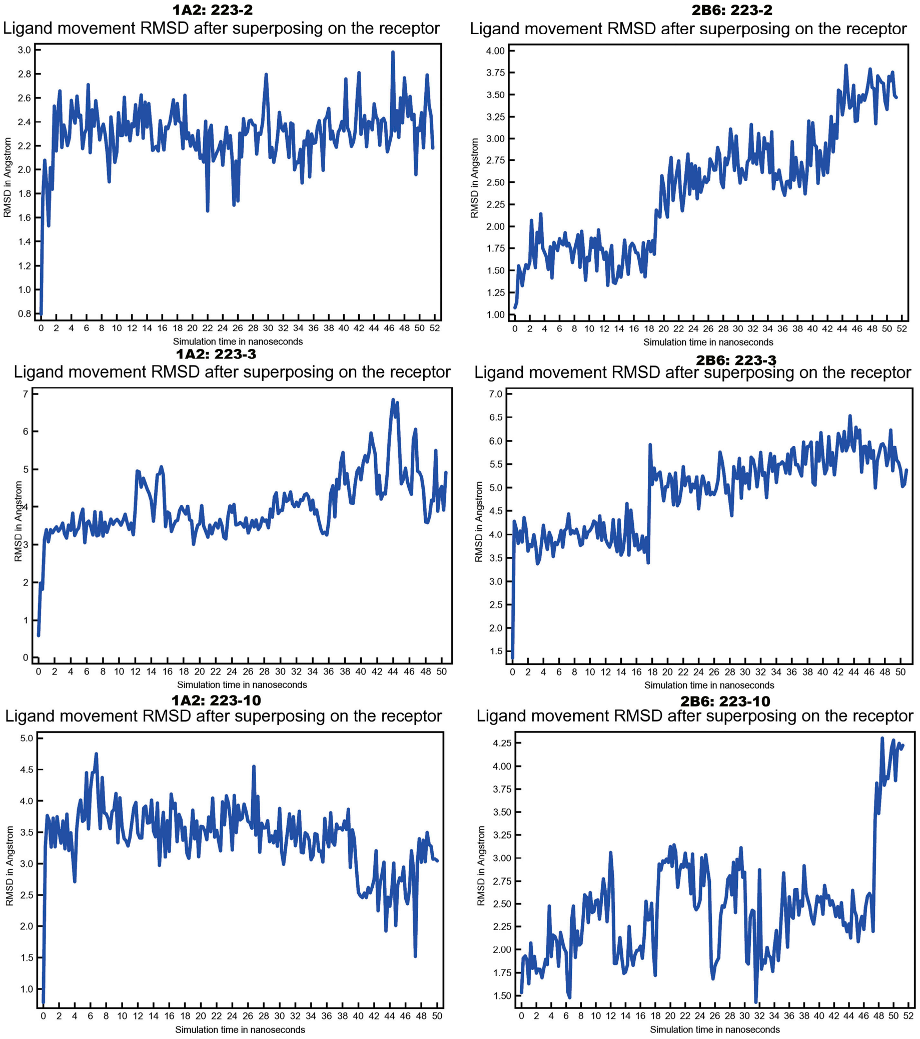

Figure S14. The TbB ligands movement RMSD after MD simulations in each of CYPs. A significant fluctuation in RMSD, indicates ligand movement in the active site pocket.

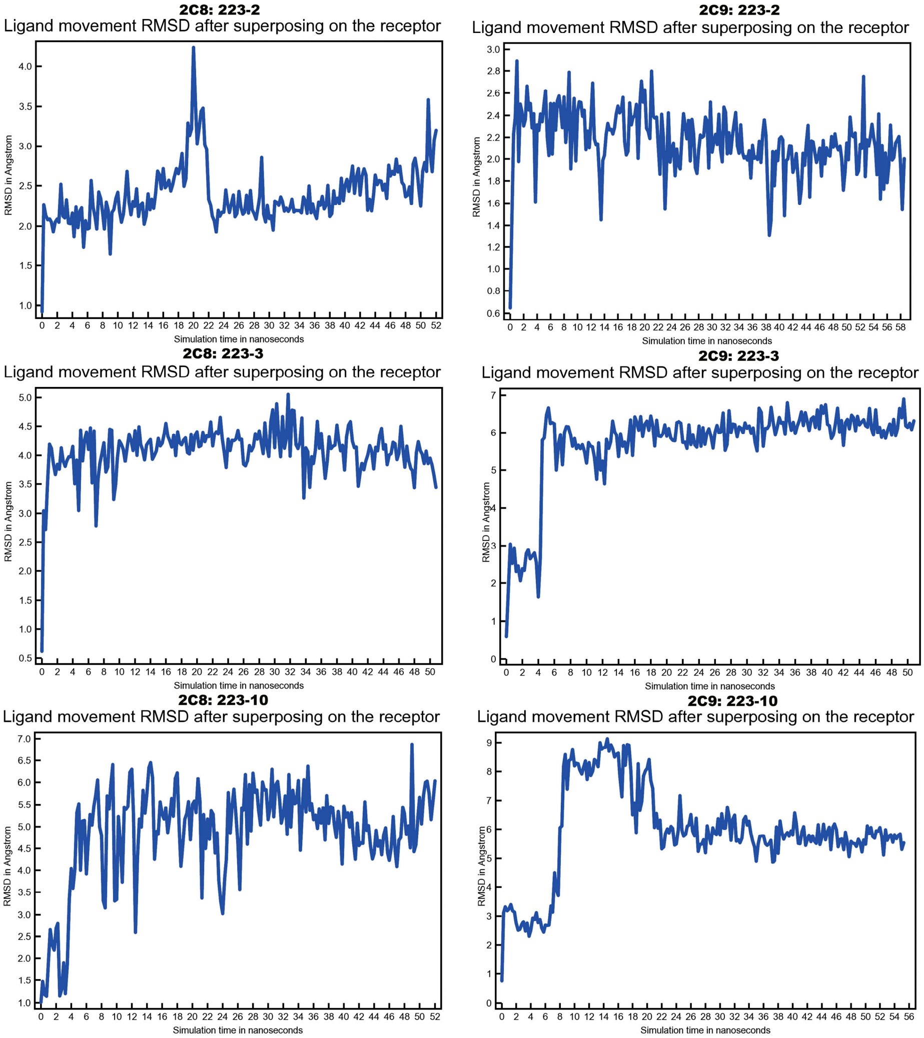

Figure S15. The TbB ligands movement RMSD after MD simulations in each of CYPs. A significant fluctuation in RMSD, indicates ligand movement in the active site pocket.

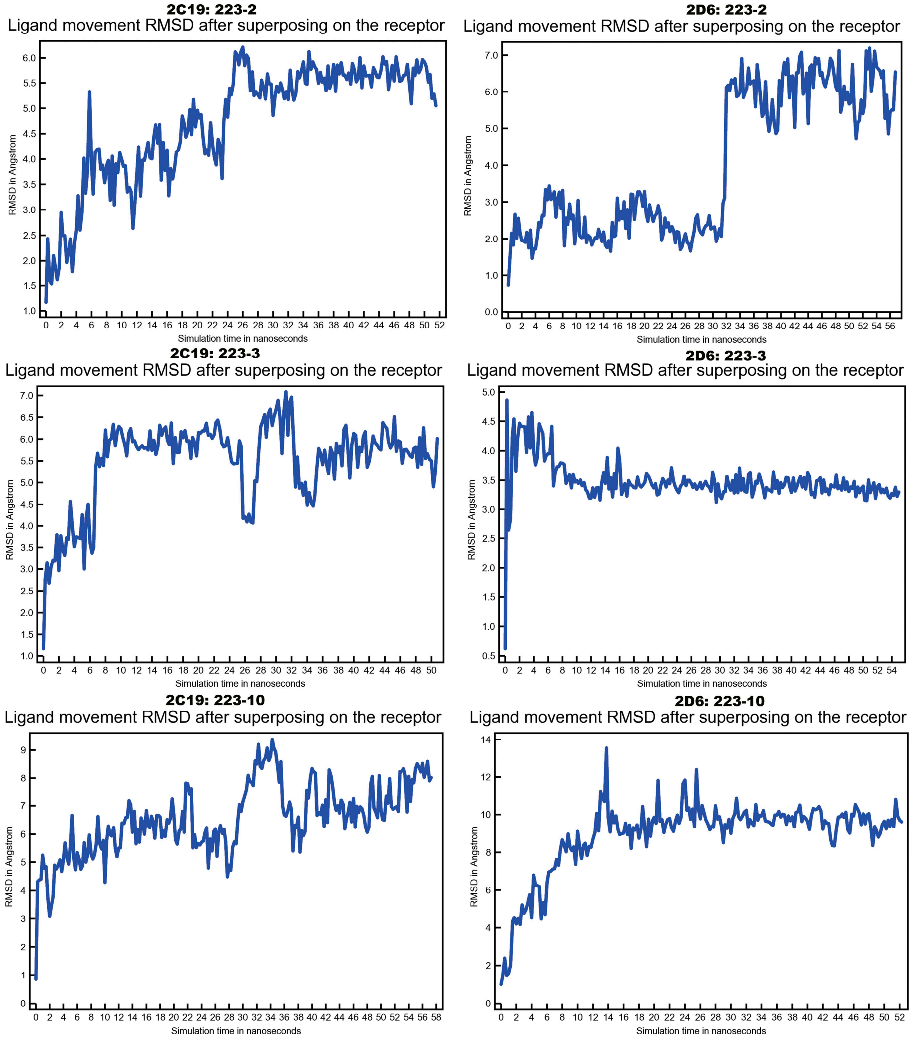

Figure S16. The TbB ligands movement RMSD after MD simulations in each of CYPs. A significant fluctuation in RMSD, indicates ligand movement in the active site pocket.

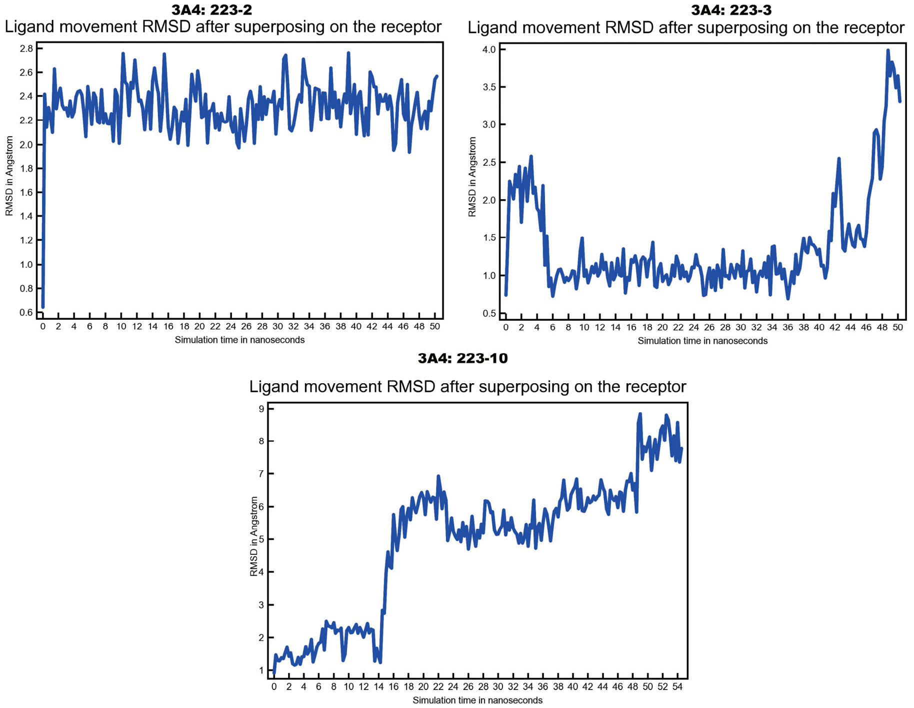

Figure S17. The TbB ligands movement RMSD after MD simulations in each of CYPs. A significant fluctuation in RMSD, indicates ligand movement in the active site pocket.

Table S4. Intrinsic clearance comparative studies with HLM and CPHH at test conc.1.0E-07 M and 1.0E-06 M respectively.

Ligands	Half-life		Clint	
	HLM	CPHH	HLM	CPHH
223-2	809	33	<115.5	29.8
223-3	75	54	<115.5	18.4
223-10	180	36	<115.5	27.8

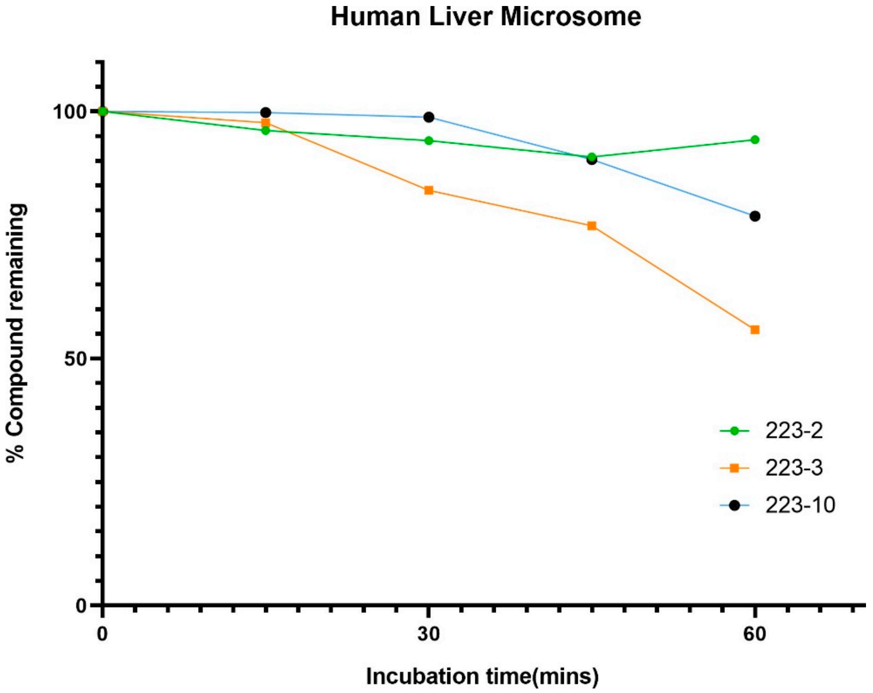

Figure S18. Metabolic stability studies of TbB ligands in HLM. More than 50% of the parent compounds remained after incubation suggesting that these ligands predominantly are stable and undergoing Phase I metabolism. (n=2).
